# Possible Synthetic Approaches for Heterobimetallic Complexes by Using *n*NHC/*tz*NHC Heteroditopic Carbene Ligands

**DOI:** 10.3390/molecules24122305

**Published:** 2019-06-21

**Authors:** Andrea Longhi, Marco Baron, Marzio Rancan, Gregorio Bottaro, Lidia Armelao, Paolo Sgarbossa, Cristina Tubaro

**Affiliations:** 1 Department of Chemical Sciences, University of Padova, via F. Marzolo 1, 35131 Padova, Italy; andrea.longhi.1@phd.unipd.it (A.L.); marco.baron@unipd.it (M.B.); lidia.armelao@unipd.it (L.A.); 2 Institute of Condensed Matter Chemistry and Technologies for Energy, National Research Council, ICMATE-CNR, c/o Department of Chemical Sciences, University of Padova, via F. Marzolo 1, 35131 Padova, Italy; marzio.rancan@cnr.it (M.R.); gregorio.bottaro@cnr.it (G.B.); 3 Department of Industrial Engineering, University of Padova, via F. Marzolo 9, 35131 Padova, Italy; paolo.sgarbossa@unipd.it

**Keywords:** gold(I) complexes, NHC ligands, *abnormal* NHC ligands, heteroditopic ligands

## Abstract

The synthesis of heterobimetallic complexes remains a synthetic challenge in the field of organometallic chemistry. A possible approach in this regard might be the use of a bidentate heteroditopic bis(carbene) ligand that combines an imidazol-2-ylidene (*n*NHC) with a 1,2,3-triazol-5-ylidene (*tz*NHC) connected by an organic spacer. The optimized strategy to heterobimetallic complexes with this type of ligand involves a 3-step procedure: (i) Coordination of the *n*NHC, functionalized with a 1,2,3-triazole ring, to a metal center; (ii) formation of the triazolium ring by alkylation of the triazole N-3; (iii) deprotonation of the *tz*NHC precursor and coordination of the second metal center. Following this procedure, a novel Au(I)-Ag(I) dinuclear complex was isolated and its properties were compared to the analogous homobimetallic Ag(I)-Ag(I) and Au(I)-Au(I) complexes. The study was completed by the determination of the molecular structures of some synthetic intermediates.

## 1. Introduction

Since the first report by Arduengo on an isolable N-heterocyclic carbene (NHC) in 1991 [1], this class of compounds has become widely popular in coordination chemistry [2,3,4]. Among the assets of NHCs as ligands, we find the high stability of their metal complexes, due to the strength of the carbene-metal bond, the possibility of easily introducing functional groups in their structure and the tuning of the steric and electronic properties of the metal center by varying the nature and the properties of the heterocyclic ring. NHC metal complexes find thus application in homogeneous catalysis [5,6,7], in the preparation of luminescent [8] and liquid crystalline materials [9] and as metallodrugs [10,11,12].

Aiming to preparing very stable complexes, an additional possibility, is the use of bidentate carbenes, enabling chelating or bridging coordination mode of the ligand [13]. In this regard, the possibility of preparing dinuclear complexes is of particular interest in the case of linearly dicoordinated d^10^ metal centers, which are known to present metal-metal interactions, the so-called metallophilic interaction. The aurophilic interaction between gold(I) centers, introduced by Schmidbaur [14], is the most famous phenomenon of this type, and its presence, together with the relativistic effects distinctive of gold, might be connected with high-quality emission properties [15,16,17].

**Scheme 1:**
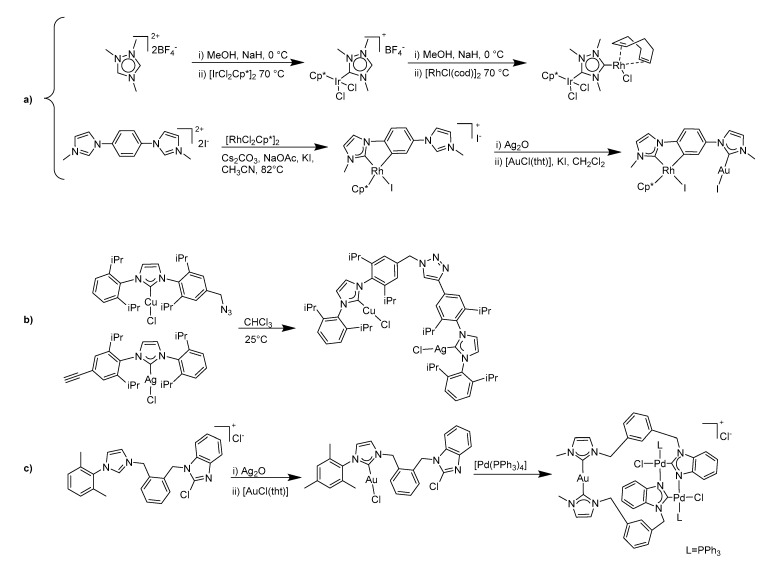
Examples of possible strategies for the isolation of heterobimetallic complexes reported in the literature and described in the text: (**a**) stepwise metalation of polycarbene ligands; (**b**) linking of metal complexes via post-functionalization of carbene ligands; (**c**) coordination of two metals via two different reactions (deprotonation of the azolium salt and oxidative addition).

Another interesting opportunity with bidentate ligands is the possibility to synthesize homo- or heterobimetallic complexes. The possibility of combining different metals in one compound is particularly fascinating in the field of homogeneous catalysis, allowing in principle cooperativity effects between metal centers with different electronic and coordination properties. Heterobimetallic catalysis is thus an emerging research area [18], whose development is, however, slowed down by a non-trivial synthetic problem, namely a straightforward and rational synthesis of heterobimetallic complexes. The synthesis and catalytic application of heterobimetallic complexes bearing NHC donors have been reviewed five years ago by Peris and Hahn [18] and more recent examples have been reported by the same authors [19,20,21,22], as well as by other ones [23,24]. Although successful synthetic approaches are present in the literature, the majority of them is not general and works only in specific conditions. For example, the stepwise metalation of polycarbene ligands has been successfully employed in several cases (Scheme 1a), although the coordination of two different metal centers is driven by the geometrical constraints of the ligand and by the different coordination geometries of the investigated metal centers [25]. In other cases, two metal complexes are linked via post-functionalization of the carbene ligands, for example by formation of a new heterocyclic ring (namely the 1,2,3-triazole) (Scheme 1b) [26]. Finally, Hahn and co-workers have recently reported a procedure based on a bis-NHC precursor in which the two heterocyclic rings can be coordinated to the two metal centers, via different reactions, i.e., deprotonation of the azolium salt and oxidative addition of the C-Cl bond (Scheme 1c) [27].

We have recently reported an *n*NHC-*tz*NHC heteroditopic ligand with a propylene linker between the two carbene donors [28]; considering the synthetic procedure to obtain the ligand precursor, we embarked on the investigation of a rational stepwise synthetic procedure for the isolation of heterobimetallic complexes with this type of ligand. A very similar *n*NHC-*tz*NHC ligand (with a shorter linker between the carbene units) was used by Cowie et al. in the synthesis of a Pd(II)/Rh(I) heterobimetallic complex in a stepwise metalation approach [29], exploiting the different acidic properties of the two azole rings [30]. They recognized indeed that the outcome of the reaction was not general, toughly depending on the used metal precursors.

Here, we report on our attempts to isolate heterobimetallic complexes, following different synthetic approaches, dealing mainly with the post-functionalization of an already coordinated carbene ligand. It can be anticipated that a novel silver(I)-gold(I) heterobimetallic complex has been isolated and characterized, together with its homobimetallic analogues. Moreover, mononuclear gold(I) complexes, intermediates in the synthetic procedure, have been fully characterized by means of ^1^H and ^13^C-NMR, ESI-MS and single-crystal X-ray structure. 

## 2. Results and Discussion

### 2.1. Synthesis of the Proligands

**Scheme 2:**
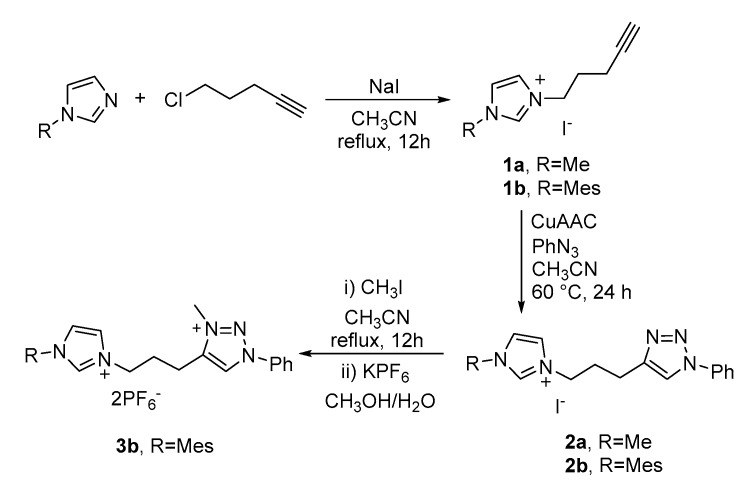
Synthesis of the imidazolium salts **1a,b**, **2a,b** and **3b**.

Alkynyl-imidazolium salts **1a**,**b** were synthesized using a slightly modified procedure reported in the literature for similar compounds [31], which involves a nucleophilic substitution reaction between an N-substituted imidazole and 5-chloro-1-pentyne in acetonitrile under reflux (Scheme 2).

Products **1a,b** were synthesized in high yield and their formation was confirmed by the ^1^H-NMR spectra (see Supplementary Material), which present the characteristic imidazolium C2-H proton at ca. 9.7 ppm. The 1,2,3-triazole moiety was then obtained via the common copper azido-alkyne 1,3-dipolar cycloaddition (CuAAC), although a stoichiometric amount of Cu(II) and sodium ascorbate was required (Scheme 2) [32]. The success of the reaction can be easily checked with the ^1^H-NMR spectra, which present multiplets in the range 7.3–7.9 ppm, associable to the phenyl ring protons, and a new signal at δ ca. 8.0 ppm attributable to the proton in position 5 of the triazole ring. Finally, the ESI-MS spectra show the fragments corresponding to the cationic part of the salt at *m*/*z* 268.05 and 372.08 for **2a** and **2b** respectively. Considering the significant amount of copper, the imidazolium salts **2a**,**b** must be carefully purified, in order to remove all the copper species, thus avoiding possible side reactions during the synthesis of the complexes (see further in the text in Section 2.2).

**Figure 1:**
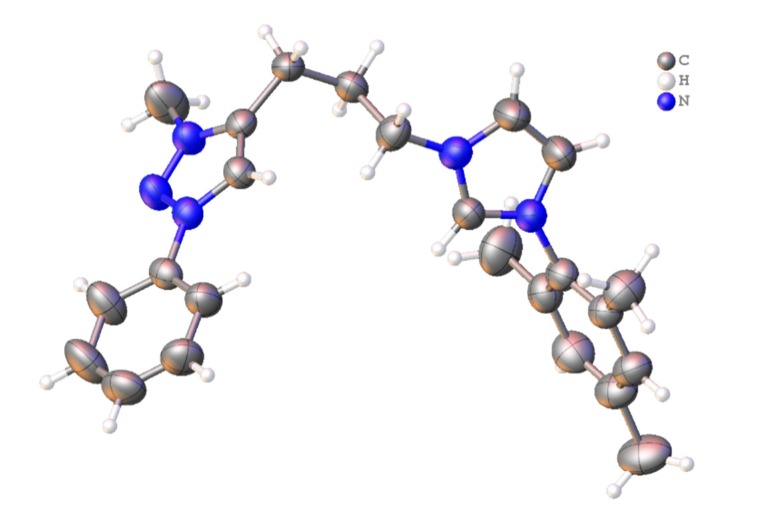
ORTEP view of the cationic part in compound **3b**. Ellipsoids are drawn at 50% probability. PF_6_^−^ anions are omitted for clarity.

Imidazolium salts **2a**,**b** are precursors of mono-*n*NHC ligands, since only the imidazolium functionality could be deprotonated to give a carbene. Only the quaternization of the N-3 triazole position with an alkyl group affords a triazolium salt, which upon deprotonation of the CH in position 5 of the ring, could give a second carbene fragment. In this view, proligand **3b** was isolated by methylation of **2b** with excess MeI in acetonitrile under reflux for 12 h, followed by I^−^/PF_6_^−^ anion exchange in a MeOH/H_2_O mixture. The ^1^H-NMR spectrum confirms the methylation of the nitrogen atom. In particular, the CH in position 5 of the triazole moves downfield at δ 8.72 ppm close to the signal of the procarbenic proton of the imidazole ring; furthermore, a singlet appears at δ 4.26 ppm, attributable to the methyl protons. The ESI-MS spectra show a peak at 532.08 *m*/*z* associated with [**3b**-PF_6_]^+^ fragment. By slow diffusion of diethyl ether into an acetonitrile solution of **3b**, some crystals suitable for X-ray single crystal diffraction analysis were obtained. The X-ray crystal structure confirms the nature of **3b** (Figure 1) as an imidazolium/triazolium salt.

### 2.2. Synthesis and Properties of Mononuclear Gold(I) Complexes

**Scheme 3:**
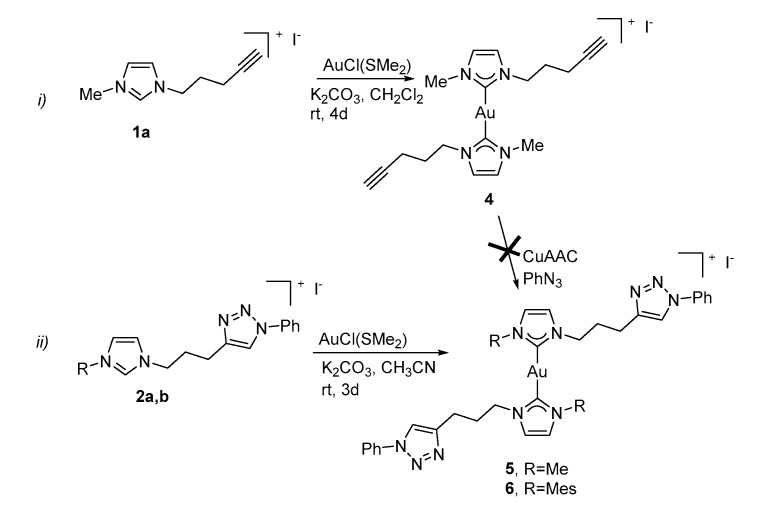
Synthesis of the gold(I) complexes **4**–**6**.

Considering the proligands described in the previous section, different procedures could be adopted for the synthesis of the gold(I) complexes, as summarized in Scheme 3. In particular, the formation of the triazole ring can occur after the coordination of the imidazol-2-ylidene to the gold center (path *i*) or before it (path *ii*).

**Figure 2:**
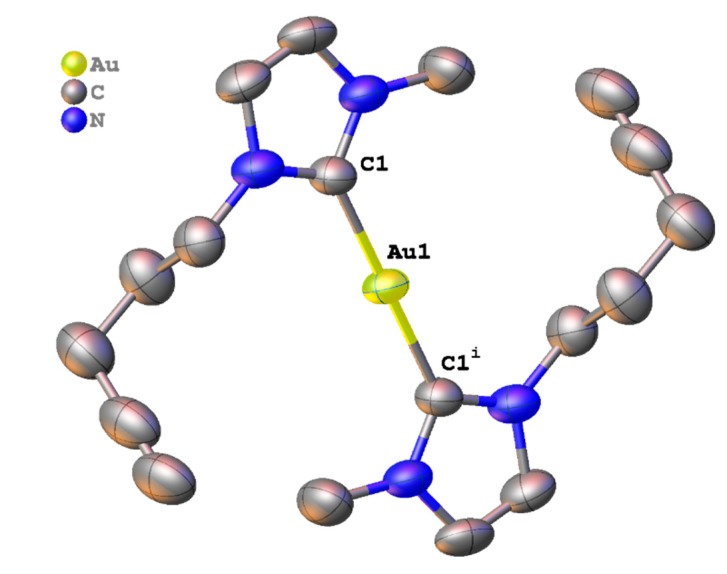
ORTEP view of the complex cation in compound **4**. Ellipsoids are drawn at 50% probability. Hydrogen atoms and iodide anion are omitted for clarity. Selected bond distances and angles: Au1—C1 2.031(6) Å and C1—Au1—C1^i^ 180°. Symmetry operation i = 1 − *x*, 1 − *y*, 1 − *z*.

Complex **4** was isolated in good yield by the reaction of the alkynyl-functionalized imidazolium salt **1a** with AuCl(SMe_2_) in a 2:1 molar ratio, in the presence of a weak base (K_2_CO_3_) under an inert atmosphere and mild conditions (room temperature in dichloromethane for 4 days). The identity of complex **4** was confirmed by ESI-MS spectra which show a peak at *m*/*z* 493.12 relative to the cationic part of the complex [AuL_2_]^+^ (L = NHC). In the ^13^C-NMR spectrum, the signal at δ 185.0 ppm is attributed to the C2-imidazole carbene coordinated to the gold(I) center [33]. The definitive support for the proposed structure comes from the single crystal X-ray diffraction analysis (Figure 2).

As expected for a d^10^ metal center, gold(I) has a linear coordination geometry. The carbene-Au(I) bond distance (2.031(6) Å) is comparable with the values observed in the literature for other [Au(NHC)_2_]X complexes [33]. The complex has an *anti* conformation, i.e., the bulkiest substituents of the two ligands lay on opposite sides with respect the C1—Au1—C1^i^ line. The alkyne functional groups in complex **4** could react with an azide to form the triazole moiety (path *i*, Scheme 3), however, in all the performed attempts, decomposition of complex **4** has been observed.

**Figure 3:**
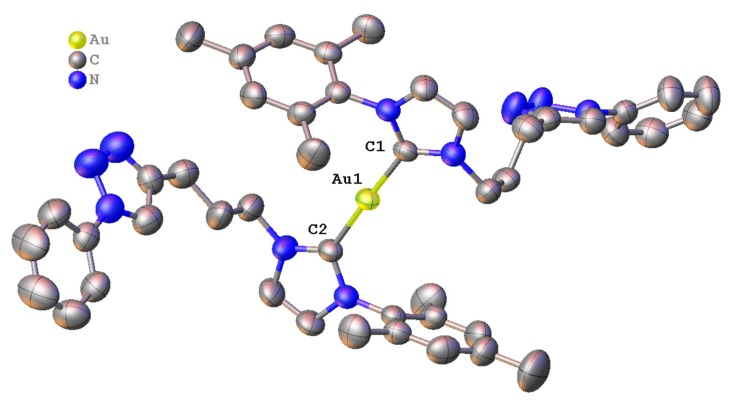
ORTEP view of the complex cation in compound **6**. Ellipsoids are drawn at 50% probability. Hydrogen atoms and iodine anion are omitted for clarity. Selected bond distances and angles: Au1—C1 2.006(4) Å, Au1—C2 2.010(4) Å and C1—Au1—C2 176.90(16)°.

For the described problems, procedure *ii)* (Scheme 2) was therefore adopted. The reaction between two equivalents of proligand **2a** (or **2b**), presenting a triazole pendant group, with AuCl(SMe_2_) in acetonitrile using K_2_CO_3_ as the base was performed (Scheme 2), affording the bis(NHC) complex **5** (or **6**) [34]. The ^13^C-NMR spectra show a signal at δ 184.8 or 184.9 ppm, for **5** and **6** respectively, in the typical range of carbene carbons coordinated to a gold(I) center [33]. The ESI-MS spectra show peaks at 731.23 or 939.27 *m*/*z*, for **5** and **6** respectively, associated with the loss of the iodide counter anion from the complex. This supports the formation of the gold(I) bis-carbene complex. Crystals of complex **6** suitable for the single crystal X-ray diffraction were obtained by slow diffusion of diethyl ether into an oily suspension of complex **6** in an acetonitrile/hexane mixture. The X-ray structure confirms the formation of the bis-carbene gold(I) complex (Figure 3).

The two carbene-Au(I) distances (2.006 and 2.010 Å, respectively) are comparable with those reported in the literature for other bis-carbene Au(I) complexes [33]. Also in this case, the two ligands adopt an *anti* conformation.

**Figure 4:**
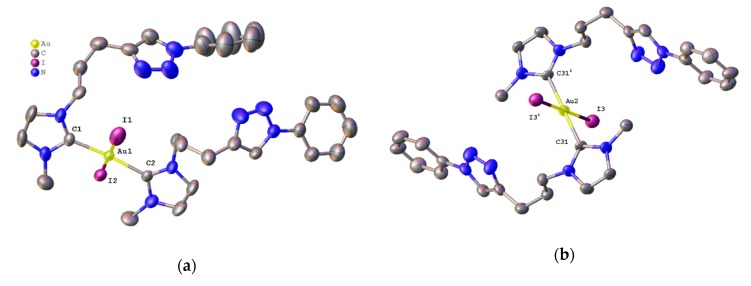
ORTEP view of the complex cation in compound **7**. Ellipsoids are drawn at 50% probability. Hydrogen atoms and uncoordinated iodide anions are omitted for clarity. Two conformational isomers are present in the unit cell: (**a**) The *syn* species, (**b**) the *anti* one. Selected bond distances and angles: Au1—C1 2.030(11) Å, Au1—C2 2.047(11) Å, Au1—I1 2.6390(10) Å, Au1—I2 2.6324(9), Au2—C31 2.050(10) Å, Au2—I3 2.6409(9) Å and C1—Au1—C2 174.3(4)°, I1—Au1—I2 179.59(4)°, C31—Au2—C31^i^ 180°, I3—Au2—I3^i^ 180.00(3)°. Symmetry operation i = 1 − *x*, 1 – *y*, 1 − *z*.

When using an impure batch of proligand **2a**, the solid isolated at the end of the synthesis was red colored, while normally the Au(I) NHC complexes are white or off-white solids. Moreover, the ^1^H-NMR spectrum of this solid shows the presence of two main sets of signals (in ca. 1/1 ratio), one of which associable to complex **5**. The nature of the second species, complex **7**, was fully clarified thanks to single crystal X-ray diffraction analysis. The compound is an Au(III) complex (Figure 4) with a square planar geometry, as expected for d^8^ metal center, where two NHC ligands and two iodide anions coordinate the metal. A third, uncoordinated, iodide anion is present for charge balance reasons. The obtained isomer is the *trans* one, as already observed in other I_2_ oxidative addition reactions to gold(I) centers [35]. 

Very interestingly, in the crystal, contrary to what found in the case of complexes **4** and **6**, two crystallographic independent conformational isomers (*syn* and *anti*) can be found. Figure 4a shows the *syn* conformation while the *anti* one is depicted in Figure 4b. The geometry of the *syn* isomer is slightly deformed from ideal angles values (for instance: C1—Au1—C2 174.3(4)°), probably because of the steric repulsion between the bulky substituents. 

**Figure 5:**
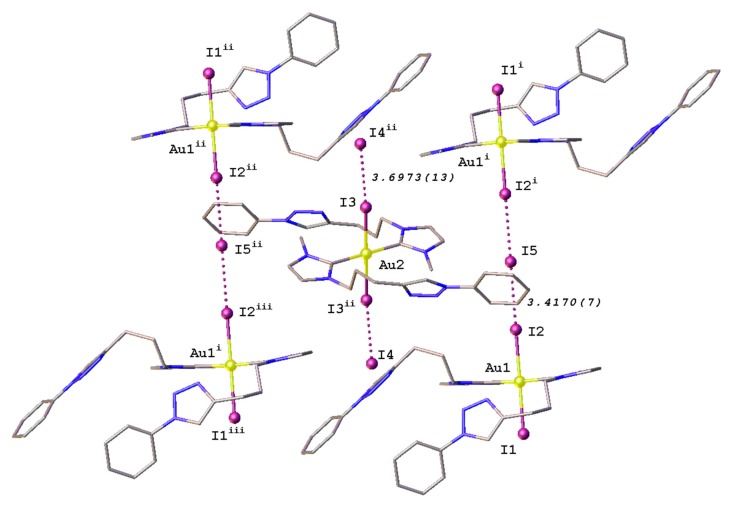
Packing in the crystal of complex **7**, with the repetition of *syn*-*anti*-*syn* layers. Symmetry operations i = −*x*, −*y*, 1 − *z*; ii = 1 − *x*, 1 − *y*, 1 − *z*; iii = 1 + *x*, 1 + *y*, *z*.

The packing in the crystal consists of the repetition of *syn*-*anti*-*syn* layers. Between each *syn* layers, there are uncoordinated iodide anions interacting with iodides bonded to the gold(III) centers forming the following connectivity I-Au-I···I···I-Au-I, with I···I distances of 3.4170(7) Å. In the case of the *anti* isomer, the interactions involving the metal and the iodide anions (coordinated and not) give connectivity of the type I···I-Au-I···I, with I···I distances of 3.6973(13) Å (Figure 5). The presence of these I···I interactions could justify the stabilization in the crystal lattice of the *syn* isomer, that should have higher energy because of the major steric repulsions between the NHC chains, when compared to the *anti* species.

The oxidation to Au(III) could be correlated to the presence of impurities of copper species still present in the ligand precursor. In fact, it is well known that Cu(II) can oxidize iodide to iodine and that iodine can then oxidize gold(I) to gold(III).

In the NMR spectra, the signals associated with complex **7** are very similar (in number and multiplicity) to those detected for complex **5**, although slightly shifted to higher ppm, as expected for a complex in which the metal center has a higher Lewis acid character. 

### 2.3. Synthesis and Properties of Dinuclear Complexes 

In view of isolating heterobimetallic complexes, the alkylation of the N-3 position of the triazole ring is required, so that upon deprotonation of C5-H, a second carbene ligand can be obtained. The stepwise methylation approach was also adopted by Barnard and coworkers for the isolation of dinuclear heterobimetallic complexes having two bridging di-*n*NHC ligands [36].

**Scheme 4:**
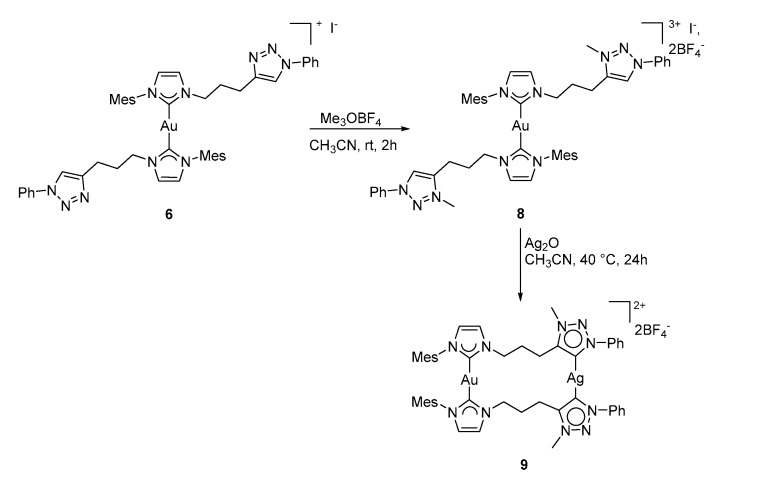
Synthesis of complexes **8** and **9**.

Complex **6** was therefore converted to complex **8** by using a strong methylating agent, the Meerwein salt Me_3_OBF_4_, in 2 h in acetonitrile solution (Scheme 4). Other methylating agents, like for example MeI do not afford any product; this is not surprising considering that also in the literature it is reported that strong alkylating agents should be used in the case of triazole rings [28]. 

The formation of complex **8** can be confirmed by the ^1^H-NMR spectrum, in which a new methyl signal at δ 4.20 ppm appears; furthermore, the triazole C5-H results deshielded from δ 8.11 to 8.74 ppm, due to the positive charge on the ring. The ^13^C-NMR spectrum shows the new CH_3_ signal at δ 38.6 ppm, without losing the carbene signal at 184.9 ppm. The ESI-MS presented the peak at *m*/*z* 1143.33 associated with the fragment [**8**-I]^+^ (loss of an iodide anion).

Complex **8** was reacted with Ag_2_O in acetonitrile, heating the mixture at 40 °C in a pressure tube for 24 h (Scheme 4). In the ^1^H-NMR spectrum of complex **9**, it is possible to observe the disappearance of the C5-H signal; this suggests the deprotonation of the triazolium ring to form the carbene ligand. The ^13^C-NMR spectrum shows two carbene signals, at 184.0 for the C_im_-Au and 165.1 for the C_tz_-Ag; the chemical shift of these signals is comparable with those reported in the literature for imidazol-2-ylidene and 1,2,3-triazol-5-ylidene ligands coordinated to gold(I) [15,33,37] and silver(I) [38,39] centers respectively. Furthermore, similar chemical shifts were observed for the previously synthesized homobimetallic Au(I)-Au(I) and Ag(I)-Ag(I) complexes having an analogous ditopic *n*NHC-*tz*NHC ligand [28]. As expected the mesoionic carbene carbon (C_tz_) has a lower chemical shift than the *normal* carbene (C_im_), indicative of superior donor properties [40,41,42]. The ESI-MS spectra show the signal at 1163.25 *m*/*z* associated with the fragment [**9**-BF_4_]^+^ and the simulated isotopic pattern clearly matches the experimental one. Complex **9** evidenced a moderate tendency to decomposition (both in solution and solid state) developing a dark color, probably due to the formation of metallic aggregates, after few days of exposure to light at ambient atmosphere. Interestingly, complex **9** might represent the precursor of others heterobimetallic complexes and we report here only the transmetalation to gold(I). The results obtained in this reaction are particularly helpful for the identification of one of the homobimetallic gold species (see further in the text) and for this reason, they will be discussed in the next paragraph.

**Scheme 5:**
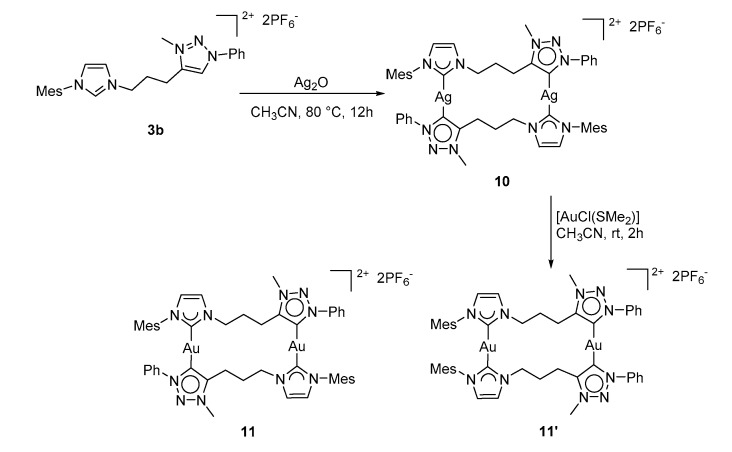
Synthesis of the dinuclear silver(I) complex **10** and of the gold(I) complexes **11** and **11′.**

To compare the properties of the heterobimetallic complex **9**, its homobimetallic silver(I) and gold(I) analogues were synthesized, following already reported procedures (Scheme 5). 

The dinuclear homobimetallic silver(I) complex **10** was isolated by the reaction of the bis(azolium) salt **3b** with silver(I) oxide in acetonitrile at 80 °C. The identity of the silver(I) complex as a dinuclear dicationic species with two bridging di(N-heterocyclic carbene) ligands has been supported by the disappearance of the two signals at ca. 8.5 ppm suggesting the deprotonation of the diazolium salt and by the ESI-MS spectrum showing the fragment [**10**-PF_6_]^+^ at 1131.04 *m*/*z*. 

Subsequently, the gold(I) complex was synthesized by transmetalation reaction between the silver complex **10** and AuCl(SMe_2_) in acetonitrile as a solvent, using an Ag:Au ratio ca. 1:1. In this case, two sets of signals are present in the ^1^H-NMR spectra in a 1:1 ratio, associable to two constitutional isomers of the gold(I) complex; in particular, we have recently reported and commented on this regard, concluding that the two isomers are associable to the head-to-head or head-to-tail coordination of the heteroditopic ligand [28]. We have labeled the crude mixture of complexes isolated at the end of the synthesis **11/11′** with the apostrophe indicating the head-to-head coordination of the ligand. In the ESI-MS spectrum, the mixture **11/11′** shows a signal at 1309.23 *m*/*z* corresponding to the loss of one PF_6_^-^ anion from the complex.

**Figure 6:**
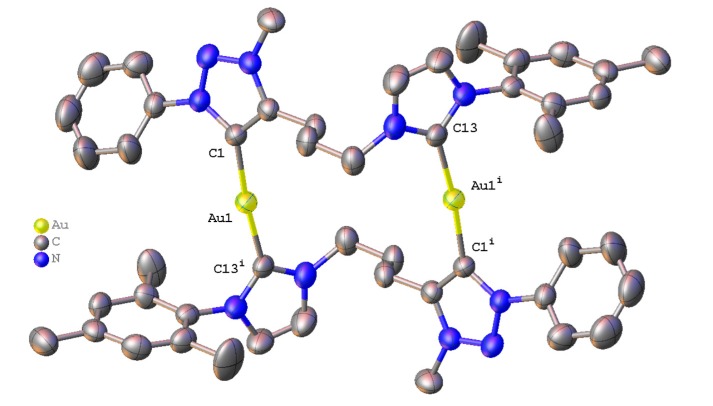
ORTEP view of the complex cation in compound **11**. Ellipsoids are drawn at 50% probability. PF_6_^-^ anions and hydrogen atoms are omitted for clarity. Selected bond distances and angles: Au1—C1 2.028(5) Å, Au1—C13^i^ 2.027(5) Å, and C1—Au1—C13^i^ 173.78(4)°. Symmetry operation i = 1 − *x*, 1 − *y*, 1 − *z*.

The gold(I) isomers could be separated by slow diffusion of diethyl ether into an acetonitrile solution of the mixture. In particular, it was possible to isolate few crystals of the less soluble isomer **11**, suitable for single crystal XRD analysis. The solved structure (Figure 6) shows the head-to-tail coordination of the ligand with a stretched conformation of the bridge. The two gold centers stand at the distance of 6.2 Å, thus the presence of aurophilic interaction can be excluded in this compound. Bond distances of the metal coordination sphere are very similar to what is found in the other Au compounds here presented, while the coordination geometry of the gold center is slightly deformed from ideal angles values (C1—Au1—C13^i^ 173.78(4)°), probably because of structural constrains imposed by the cyclic structure.

**Figure 7:**
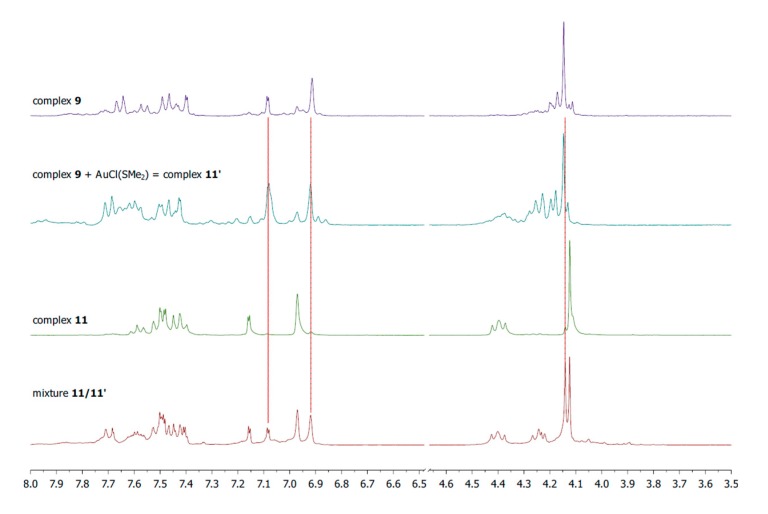
^1^H-NMR spectra in deuterated acetonitrile of complex **9** (purple spectrum), complex **9** + excess of AuCl(SMe_2_) (blue spectrum), complex **11** (green spectrum) and mixture **11/11’** in 1/1 ratio, isolated with the procedure described in Scheme 5 (red spectrum).

An indirect proof of the identity of complex **11′** as dinuclear gold(I) complex with the two bridging dicarbene ligands coordinated in head-to-head coordination, comes from the Ag-Au transmetalation reaction starting from complex **9**, in which the head-to-head coordination of the ligand is imposed by the synthetic procedure. We added a slight excess of AuCl(SMe_2_) to the solution of complex **9** in deuterated acetonitrile and followed the reaction via ^1^H-NMR spectra. The transmetalation reaction is accompanied by the formation of an off-white precipitate (the insoluble AgCl) and, as evidenced by the spectra reported in Figure 7, the main product of the reaction is complex **11′**. In fact, the diagnostic signals of complex **11′** (indicated with a red line) are practically coincident with those of the species present in the NMR reaction mixture. Moreover, the chemical shifts of the signals associated with complex **11′** are very close to those of complex **9**, that should present a very similar structure. This is not surprising because it is well known that the substitution of Ag atoms with Au ones in NHC complexes has a negligible influence in the position of the proton NMR signals.

### 2.4. Luminescence Properties

**Figure 8:**
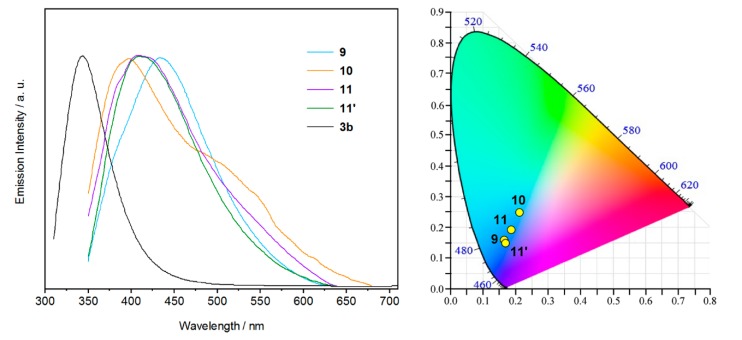
PL spectra of ligand **3b** and complexes **9**, **10**, **11** and **11′** obtained exciting the sample with UV light at 330 nm. The color coordinates calculated from the emission spectra are reported in the CIE1931 chromaticity diagram.

The room temperature emission spectra of the **3b** ligand and of **9**, **10**, **11** and **11′** complexes were obtained from films prepared drop-casting acetonitrile solutions of the different compounds onto silica slides (Figure 8). The photoluminescence spectra of the complexes are red-shifted compared with that of the diazolium salt **3b** (emission λ_max_ 335 nm) precursor of the *n*NHC-*tz*NHC ligand. The observed shift is ascribed to the metal coordination which causes a perturbation on the molecular orbitals as often happen in this kind of noble metal complexes [15,28,43,44]. All the dinuclear complexes have emission maxima in the blue-region between 400–440 nm (λ_max_ = 395 for **9**; 412 for **11** and **11′** and 435 nm for **10**). Besides for the position of the emission maxima, the PL spectra of **9**, **10**, **11** and **11′** show a different broadening in the long wavelength side that induce a color shift toward the white region of the CIE1931 chromaticity diagram. 

The spectra of **11** and **11′** are similar to those of the analogue homobimetallic complex [Au_2_{MeIm(CH_2_)_3_*tz*NHC}_2_](PF_6_)_2_ having a methyl as wingtip group on the *N*-imidazole ring instead of a mesityl one [28]. Moreover, it is interesting to underline that the emission λ_max_ in complex **11** is blue-shifted compared to that of the complex [Au_2_{MeIm(CH_2_)_3_*tz*NHC}_2_](PF_6_)_2_ and this can be ascribed to the absence of aurophilic interaction in complex **11**. The intramolecular Au···Au distance in **11** is in fact 6.2 Å longer than that observed in [Au_2_{MeIm(CH_2_)_3_*tz*NHC}_2_](PF_6_)_2_ (3.068 Å).

## 3. Materials and Methods

All manipulations and synthesis were carried out using standard Schlenk techniques under inert atmosphere of argon, or in a dry box MBraun Labmaster operating in dinitrogen atmosphere. The reagents and solvents were purchased as high-purity products and used as received. 1-mesityl-1H-imidazole was synthesized according to a procedure published in the literature [45]. NMR spectra were recorded at room temperature (298 K) on a Bruker Avance 300 MHz spectrometer (300.1 MHz for ^1^H, 75.5 MHz for ^13^C); chemical shifts (δ) are reported in ppm relative to the residual solvent signals. The ESI-MS analysis was performed using a Thermo-Finnigan LCQ-Duo (San Jose, CA, USA) coperating in positive mode. The elemental analysis was carried out with a Thermo Scientific FLASH 2000 apparatus.

### 3.1. Synthesis of the Imidazolium Proligands 

#### 3.1.1. Synthesis of the Alkynyl Imidazolium Salts **1a** and **1b**

The proper N-substituted imidazole (3.0 mmol; 0.24 mL of *N*-methylimidazole or 0.56 g of *N*-mesitylimidazole), NaI (0.51 g, 3.4 mmol), 5-chloro-1-pentyne (0.5 mL, 4.7 mmol) and 10 mL of CH_3_CN were introduced in a round bottom flask. The mixture was stirred under reflux for 24 h, then the solvent was removed under vacuum and the residue was treated with 10 mL of dichloromethane. The obtained mixture was filtered over Celite and the solution evaporated to dryness. **1a**. Orange solid (yield 70%). ^1^H-NMR (300 MHz, CDCl_3_) δ 9.85 (s, 1H, NCHN), 7.58 (s, 1H, CH_im_), 7.55 (s, 1H, CH_im_), 4.48 (m, 2H, NCH_2_), 4.09 (s, 3H, NCH_3_), 2.31 (m, 2H, CH_2_C), 2.16 (m, 2H, CH_2_), 2.10 (m, 1H, CH_alkyne_). ^13^C-NMR (75 MHz, CDCl_3_) δ 136.9 (NCHN), 123.8 (CH_im_), 122.7 (CH_im_), 81.5 (C_alkyne_), 71.2 (C_alkyne_), 48.7 (NCH_2_), 37.3 (NCH_3_), 28.6 (CH_2_), 15.5 (CH_2_). 

**1b**. Brown oil (yield 99%). ^1^H-NMR (300 MHz, CD_3_CN) δ 9.66 (s, 1H, NCHN), 8.08 (s, 1H, CH_im_), 7.66 (s, 1H, CH_im_), 7.06 (s, 2H, CH_Mesityl_), 4.53 (m, 2H, NCH_2_), 2.41 (m, 1H, CH_alkyne_), 2.26 (m, 2H, CH_2_C), 2.23 (s, 3H, Me_Mesityl_), 2.14 (m, 2H, CH_2_), 2.02 (s, 6H, Me_Mesityl_). ^13^C-NMR (75 MHz, CD_3_CN) δ 141.4 (C_Mesityl_), 137.8 (NCHN), 135.1 (C_Mesityl_), 131.5 (C_Mesityl_), 130.1 (C_Mesityl_), 124.6 (CH_im_), 124.1 (CH_im_), 82.8 (C_alkyne_), 71.8 (C_alkyne_), 49.4 (NCH_2_), 29.2 (CH_2_), 21.3 (Me_Mesityl_), 18.1 (Me_Mesityl_), 15.8 (CH_2_).

#### 3.1.2. Synthesis of Proligands **2a** and **2b**

CuSO_4_·5H_2_O (0.10 g, 0.4 mmol), sodium ascorbate (0.12 g, 0.6 mmol) and 7 mL of CH_3_CN were introduced in an ACE pressure tube. The mixture was stirred at room temperature for half an hour and then the proper alkynyl-imidazolium salt **1a** or **1b** (0.4 mmol) was added; the resulting mixture was further stirred at room temperature for one hour. Finally, phenylazide (0.80 mL of a 0.5 M solution in t-butylmethylether, 0.4 mmol) was added and the mixture was left under stirring at 60 °C for 24 h. The resulting mixture was filtered and the addition of KI (0.13 g, 0.8 mmol) to the filtrate caused extra precipitation of a white solid, which was filtered. The filtrate was evaporated under vacuum to remove the volatiles. The oily residue was dissolved in dichloromethane (ca. 15 mL), washed with aqueous ammonia (15 mL), water (5 mL) and finally dried over Na_2_SO_4_. Removal of the volatiles afforded the desired products. **2a**. Slightly yellow oil. ^1^H-NMR (300 MHz, CDCl_3_) δ 8.86 (s, 1H, NCHN), 8.01 (s, 1H, CH_tz_), 7.75 (d, *J* = 7.8 Hz, 2H, ArH), 7.60–7.34 (m, 4H, ArH + CH_im_), 7.19 (s, 1H, CH_im_), 4.33 (m, 2H, NCH_2_), 3.93 (s, 3H, NCH_3_), 2.87 (m, 2H, CH_2_C), 2.38 (m, 2H, CH_2_). ESI-MS (positive ions, CH_3_CN): *m*/*z* 268.05 ([**2a**-I]^+^). **2b**. Very hygroscopic yellow solid (yield 55%). ^1^H-NMR (300 MHz, CD_3_CN) δ 9.20 (s, 1H, NCHN), 8.35 (s, 1H, CH_tz_), 7.83 (m, 3H, ArH + CH_im_), 7.63–7.34 (m, 4H, ArH + CH_im_), 7.07 (s, 2H, CH_Mesityl_), 4.47 (m, 2H, NCH_2_), 2.85 (m, 2H, CH_2_C), 2.43 (m, 2H, CH_2_), 2.38 (s, 3H, Me_Mesityl_), 2.04 (s, 6H, Me_Mesityl_). ^13^C-NMR (75 MHz, CD_3_CN) δ 147.5 (C_tz_), 142.0 (C_Mesityl_), 138.0 and 137.8 (NCHN + C_Mesityl_), 135.6 (C_Mesityl_), 131.8 (C_Mesityl_), 130.7 (ArH), 130.3 (CH_Mesityl_), 129.4 (ArH), 124.8 (CH_im_), 124.2 (CH_im_), 121.6 (CH_tz_), 121.0 (ArH), 50.0 (NCH_2_), 29.9 (CH_2_), 22.6 (CH_2_C), 21.1 (Me_Mesityl_), 17.6 (Me_Mesityl_). ESI-MS (positive ions, CH_3_CN): *m*/*z* 372.08 (100% [**2b**-I]^+^).

#### 3.1.3. Synthesis of Proligand **3b**

MeI (0.2 mL, 3.4 mmol) was added to an acetonitrile solution (5 mL) of imidazolium salt **2b** (0.17 g, 0.34 mmol); the resulting solution was stirred under reflux for 12 h. The solvent was then removed under vacuum giving a dark brown solid, which was dissolved in MeOH (5 mL) and treated with a solution of KPF_6_ (0.32 g, 1.7 mmol) in water (5 mL). The formed brownish precipitate was filtered and dried under vacuum. Brownish solid (yield 58%) ^1^H-NMR (300 MHz, CD_3_CN) δ 8.76 and 8.72 (2s, 2H, NCHN+CH_tz_), 7.88 (m, 2H, ArH), 7.73 (m, 4H, ArH+CH_im_), 7.51 (s, 1H, CH_im_), 7.13 (s, 2H, CH_Mesityl_), 4.42 (m, 2H, NCH_2_), 4.26 (s, 3H, Me_tz_), 2.97 (m, 2H, CH_2_C), 2.42 (m, 2H, CH_2_), 2.36 (s, 3H, Me_Mesityl_), 2.06 (s, 6H, Me_Mesityl_). ^13^C-NMR (75 MHz, CD_3_CN) δ 144.7 (C_tz_), 142.3 (C_Mesityl_), 137.4 (NCHN), 135.7 (Ar and C_Mesityl_), 132.9 (ArH), 131.8 (C_Mesityl_), 131.5 (ArH), 130.4 (CH_Mesityl_), 127.7 (CH_tz_), 125.3 (CH_im_), 124.2 (CH_im_), 122.5 (ArH), 49.7 (NCH_2_), 39.0 (Me_tz_), 27.7 (CH_2_C), 21.1 and 21.0 (CH_2_ + Me_Mesityl_), 17.5 (Me_Mesityl_). ESI-MS (positive ions, CH_3_CN): *m*/*z* 372.14 ([**3b**-2PF_6_-CH_3_]^+^), 532.08 ([**3b**-PF_6_]^+^). Colourless needle-like crystals were obtained by slow diffusion of diethyl ether into an acetonitrile solution of the salt.

### 3.2. Synthesis of the Gold(I) Mononuclear Complexes

#### 3.2.1. Synthesis of Complex **4**

In a round bottom flask were introduced K_2_CO_3_ (2.3 g, 16 mmol), 1-methyl-3-(pent-4-yn-1yl)-1*H*-imidazol-3-ium iodide (0.19 g, 0.68 mmol), AuCl(SMe_2_) (0.10 g, 0.34 mmol) and 50 mL of dichloromethane. The mixture was left under stirring for four days at room temperature and then filtered. The filtrate was concentrated under reduced pressure to ca. 1–2 mL, then the addition of diethyl ether causes the formation of a white solid that was filtered. White solid (yield 60%). Anal. Calcd for C_18_H_24_N_4_AuI: C 34.85, H 3.90, N 9.03%. Found: C 34.44, H 4.11, N 8.53%. ^1^H-NMR (300 MHz, CD_3_CN) δ 7.22 (d, *J* = 1.8 Hz, 1H, CH_im_), 7.18 (d, *J* = 1.8 Hz, 1H, CH_im_), 4.30 (t, *J* = 7.0 Hz, 2H, NCH_2_), 3.85 (s, 3H, NCH_3_), 2.23 (m, 3H, CH_2_ + CH), 2.07 (m, 2H, CH_2_). ^13^C-NMR (75 MHz, CD_3_CN) δ 185.0 (CAu), 124.0 (CH_im_), 122.5 (CH_im_), 83.6 (C_alkyne_), 71.0 (C_alkyne_), 50.4 (NCH_2_), 38.5 (NCH_3_), 30.8 (CH_2_), 15.9 (CH_2_). ESI-MS (positive ions, CH_3_CN): *m*/*z* 493.12 (100%, [**4**-I]^+^). Colourless crystals were obtained by slow diffusion of diethyl ether in a solution of the complex in acetonitrile. 

#### 3.2.2. Synthesis of Complex **5** and Characterization of Complex **7**

In a round bottom flask were introduced proligand **2a** (0.11 g, 0.28 mmol), K_2_CO_3_ (1.24 g, 8.97 mmol), AuCl(SMe_2_) (0.042 g, 0.14 mmol) and 10 mL of acetonitrile; the mixture was left under stirring for 3 days and then filtered to remove the inorganic salts. The volatiles were evaporated, and the yellow solid was recrystallized from acetonitrile/diethyl ether. ^1^H-NMR (300 MHz, CD_3_CN) δ 8.19 (s, 1H, CH_tz_), 7.70 (m, 2H, ArH), 7.54–7.35 (m, 3H, ArH), 7.20 (d, *J* = 1.8 Hz, 1H, CH_im_), 7.09 (d, *J* = 1.8 Hz, 1H, CH_im_), 4.22 (t, *J* = 7.0 Hz, 2H, NCH_2_), 3.73 (s, 3H, NCH_3_), 2.73 (t, *J* = 7.0 Hz, 2H, CH_2_), 2.27 (m, 2H, CH_2_). ^13^C-NMR (75 MHz, CD_3_CN) δ 184.8 (CAu), 147.8 (C_tz_), 137.9 (Ar), 130.7 (ArH), 129.2 (ArH), 123.9 (CH_im_), 122.2 (CH_im_), 121.1 (CH_tz_), 120.6 (ArH), 50.6 (NCH_2_), 38.4 (NCH_3_), 30.8 (CH_2_), 22.6 (CH_2_C). ESI-MS (positive ions, CH_3_CN): *m*/*z* 731.23 (100%, [**5**-I]^+^). 

The first attempt for the synthesis of complex **5** was performed for 4 days with a sample of proligand **2a** containing some copper impurities. The ^1^H-NMR spectrum of the solid isolated from this attempt shows the presence of two sets of signals, attributed to complexes **5** and **7** in a ratio ca. 60(**5**):40(**7**). The identity of complex **7** was clarified by X-ray diffraction analysis of some crystals obtained from the acetonitrile/diethyl ether mother liquor of the final filtration. NMR signals of **7** have been assigned in the ^1^H-NMR of the crude mixture by exclusion of the signals attributed to **5**. ^1^H-NMR (300 MHz, CD_3_CN) δ 8.11 (s, 1H, CH_tz_), 7.81 (m, 2H, ArH), 7.60–7.30* (m, 3H, ArH), 7.20 (d, 1H, CH_Im_), 7.11 (d, 1H, CH_Im_), 4.25* (m, 2H, NCH_2_), 3.76 (s, CH_3_), 2.78* (m, 2H, CH_2_), 2.25* (m, 2H, CH_2_). * signals superimposed with those of complex **5**. 

#### 3.2.3. Synthesis of Complex **6**

In a round bottom flask were introduced K_2_CO_3_ (1.5 g, 11 mmol), proligand **2b** (0.23 g, 0.45 mmol), AuCl(SMe_2_) (0.065 g, 0.22 mmol) and 10 mL of acetonitrile. The mixture was left under stirring for 2 days at room temperature and then filtered. The volatiles were evaporated from the filtrate and the white residue was recrystallized from acetonitrile/diethyl ether, filtered and dried under vacuum. Off-white solid (yield 74%). ^1^H-NMR (300 MHz, CD_3_CN) δ 8.11 (s, 1H, CH_tz_), 7.77 (d, *J* = 7.4 Hz, 2H, ArH), 7.55 (m, 2H, ArH), 7.46 (m, 1H, ArH), 7.43 (d, *J* = 1.8 Hz, 1H, CH_im_), 7.07 (d, *J* = 1.8 Hz, 1H, CH_im_), 6.94 (s, 2H, CH_Mesityl_), 4.15 (m, 2H, NCH_2_), 2.64 (m, 2H, CH_2_C), 2.31 (s, 3H, Me_Mesityl_), 1.77 (s, 6H, Me_Mesityl_), the missing CH_2_ signal is covered by the signal of water (s, 2.23). ^13^C-NMR (75 MHz, CD_3_CN) δ 184.9 (CAu), 148.0 (C_tz_), 140.4 (C_Mesityl_), 138.1 (CAr), 135.9 (C_Mesityl_), 130.7 (ArH), 130.6 (C_Mesityl_), 129.8 (CH_Mesityl_), 129.4 (ArH), 123.8 (CH_im_), 123.1 (CH_im_), 121.0 (ArH), 120.9 (CH_tz_), 51.0 (NCH_2_), 31.4 (CH_2_), 23.0 (CH_2_C), 21.1 (Me_Mesityl_), 17.6 (Me_Mesityl_). ESI-MS (positive ions, CH_3_CN): *m*/*z* 939.27 (100%, [**6**-I]^+^). Yellowish crystals were obtained by slow diffusion of diethyl ether into a solution of the complex in an acetonitrile/hexane mixture. 

#### 3.2.4. Synthesis of Complex **8** by Methylation of Complex **6**

Complex **6** (0.15 g, 0.14 mmol) was introduced in a round bottom flask and then transferred in a dry box, where the solid was dissolved in 3 mL of dry acetonitrile. Solid Me_3_OBF_4_ (0.047 g, 0.32 mmol) was added to the solution of complex **6** and the mixture was left stirring for 2 h at room temperature. When the reaction was completed, the volume of acetonitrile was reduced under vacuum. The opaque yellow solution was filtered; the addition of diethyl ether gives an off-white solid that deposits at the bottom of the flask and forms gradually a yellowish oil. The ether was removed and the oil was treated under vacuum forming a white solid (yield 99%). ^1^H-NMR (300 MHz, CD_3_CN) δ 8.74 (s, 1H, CH_tz_), 7.87 (m, 2H, ArH), 7.69 (m, 3H, ArH), 7.50 (d, *J* = 1.9 Hz, 1H, CH_im_), 7.12 (d, *J* = 1.9 Hz, 1H, CH_im_), 6.99 (s, 2H, CH_Mesityl_), 4.32 (m, 2H, NCH_2_), 4.20 (s, 3H, NCH_3_), 2.85 (m, 2H, CH_2_C), 2.38 (s, 3H, Me_Mesityl_), 2.33 (m, 2H, CH_2_), 1.78 (s, 6H, Me_Mesityl_). ^13^C-NMR (75 MHz, CD_3_CN) δ 184.9 (CAu), 145.1 (C_tz_), 140.4 (C_Mesityl_), 135.8 (C_Mesityl_) 135.7 (CAr), 132.8 (ArH), 131.4 (ArH), 131.2 (C_Mesityl_), 129.9 (CH_Mesityl_), 127.4 (CH_tz_), 124.3 (CH_im_), 123.0 (CH_im_), 122.4 (ArH), 50.5 (NCH_2_), 38.6 (NCH_3_), 28.9 (CH_2_), 21.2 and 21.0 (CH_2_C+Me_Mesityl_), 17.6 (Me_Mesityl_). ESI-MS (positive ions, CH_3_CN): *m*/*z* 1143.33 ([**8**-I]^+^), 323.36 ([**8**-2BF_4_-I]^3+^).

### 3.3. Synthesis of the Dinuclear Complexes with Bridging Dicarbene Ligands

#### 3.3.1. Synthesis of Complex **9**

Complex **8** (0.17 g, 0.14 mmol), Ag_2_O (0.072 g, 0.31 mmol) and 5 mL of acetonitrile were introduced in an ACE pressure tube, that was then closed and the mixture was heated at 40 °C for 24 h. Then the mixture was filtered using a PTFE filter (0.45 μm) and a syringe. The solvent was finally removed under vacuum and the flask transferred in a dry box; the residue was dissolved in the minimum amount of dry acetonitrile and diethyl ether was added, affording the precipitation of a white solid. Unfortunately, the solid darkens with time. ^1^H-NMR (300 MHz, CD_3_CN) δ 7.90–7.34 (m, 5H, ArH + CH_im_), 7.08 (d, *J* = 1.8 Hz, 1H, CH_im_), 6.91 (s, 2H, CH_Mesityl_), 4.16 (m, 5H, NCH_2_ + NCH_3_), 2.80 (m, 2H, CH_2_C), 2.35 (s, 3H, Me_Mesityl_), 2.22 (m, 2H, CH_2_C), 1.67 (s, 6H, Me_Mesityl_). ^13^C-NMR (75 MHz, CD_3_CN) δ 184.0 (C_im_Au), 165.1 (C_tz_Au), 148.7 (C_tz_), 141.0 (CAr), 140.3 (C_Mesityl_), 135.7 (C_Mesityl_), 131.3 (ArH), 130.6 (ArH), 129.9 (C_Mesityl_), 129.7 (CH_Mesityl_), 124.5 (ArH), 123.9 (CH_im_), 123.3 (CH_im_), 50.5 (NCH_2_), 37.2 (NCH_3_), 30.6 (CH_2_), 22.7 (CH_2_C), 21.1 (Me_Mesityl_), 17.5 (Me_Mesityl_). ESI-MS (positive ions, CH_3_CN): *m*/*z* 1161 and 1163.25 (100% [**5**-BF_4_]^+^). 

#### 3.3.2. Synthesis of Complex **10**

Proligand **3b** (0.083 g, 0.15 mmol) and Ag_2_O (0.095 g, 0.41 mmol) were introduced in a Schlenk tube under inert atmosphere and CH_3_CN (5 mL) was added. The dark mixture was left under stirring for one night at 80 °C. The mixture was filtered with a PTFE filter (0.45 μm) with a syringe, obtaining a clear solution; evaporation of volatiles affords an off-white solid, which darkens with time. ^1^H-NMR (300 MHz, CD_3_CN) δ 7.63–7.31 (m, 6H, ArH + CH_im_), 7.14 (s, 1H, CH_im_), 6.94 (s, 2H, CH_Mesityl_), 4.13 (br, 5H, NCH_2_ + NCH_3_), 2.78 (m, 2H, CH_2_), 2.31 (s, 3H, Me_Mesityl_), 2.22 (m, 2H, CH_2_C), 1.75 (br, 6H, Me_Mesityl_). ^13^C-NMR (75 MHz, CD_3_CN) δ 148.7 (C_tz_), 140.9 (C_Ar_), 140.3 (C_Mesityl_), 136.5 (C_Mesityl_), 135.8 (ArH), 131.2 (ArH), 130.6 (ArH), 130.0 (C_Mesityl_), 129.9 (CH_Mesityl_), 124.2 (CH_im_), 122.9 (CH_im_), 51.0 (NCH_2_), 37.3 (NCH_3_), 30.8 (CH_2_), 22.7 (CH_2_C), 21.0 (Me_Mesityl_), 17.7 (Me_Mesityl_). C_tz_Ag carbene signal was detected in the 2D ^13^C, ^1^H HMBC-NMR spectra at 175.7 ppm; C_im_Au not detected. ESI-MS (positive ions, CH_3_CN): *m*/*z* 492.21 ([Ag(diNHC)]^+^), 1131.04 ([**10**-PF_6_]^+^) with diNHC = *n*NHC-*tz*NHC. 

#### 3.3.3. Synthesis of Complexes **11**/**11′**

Proligand **3b** (0.083 g, 0.15 mmol) and Ag_2_O (0.095 g, 0.41 mmol), were introduced in a Schlenk tube and, after making an inert atmosphere, CH_3_CN (5 mL) was added. The dark mixture was left under stirring for one night at 80 °C. Precursor AuCl(SMe_2_) (0.036 g, 0.12 mmol) was then added to the mixture and a white-purplish solid formed. After two hours stirring, the mixture was filtered with a PTFE filter (0.45 μm) with a syringe, obtaining a clear solution; evaporation of volatiles affords an off-white solid; the residue was dissolved in the minimum amount of dry acetonitrile and diethyl ether was added, affording the precipitation of a white solid (yield 60%). The ^1^H-NMR shows two main species presents in roughly the same quantity, which are two isomeric forms of the gold complex (**11**/**11′**). ESI-MS (positive ions, CH_3_CN): *m*/*z* 1309.18 ([**11**/**11′**-PF_6_]^+^). **11**. ^1^H-NMR (300 MHz, CD_3_CN) δ 7.70–7.30 (m, 6H, ArH + CH_im_), 7.16 (d, *J* = 1.8 Hz, 1H, CH_im_), 6.97 (s, 2H, CH_Mesityl_), 4.40 (m, 2H, NCH_2_), 4.12 (s, 3H, NCH_3_), 2.89 (m, 2H, CH_2_C), 2.38 (m, 2H, CH_2_), 2.31 (s, 3H, Me_Mesityl_), 1.86 (s, 6H, Me_Mesityl_). ^13^C-NMR (75 MHz, CD_3_CN) δ 185.3 (C_im_Au), 169.8 (C_tz_Au), 148.5 (C_tz_), 140.5 (C_Mesityl_), 139.7 (C_Ar_), 135.7 (C_Mesityl_), 131.2 (ArH), 130.5 (ArH), 130.0 (CH_Mesityl_), 125.1 (ArH), 124.3 (C_Mesityl_), 124.1 (CH_im_), 122.7 (CH_im_), 51.1 (NCH_2_), 37.8 (NCH_3_), 30.7 (CH_2_), 23.0 (CH_2_C), 21.0 (Me_Mesityl_), 17.8 (Me_Mesityl_). **11′**. ^1^H-NMR (300 MHz, CD_3_CN) δ 7.80–7.34 (m, 6H, ArH and CH_im_), 7.08 (d, *J* = 1.8 Hz, 1H, CH_im_), 6.92 (s, 2H, CH_Mesityl_), 4.24 (m, 2H, NCH_2_), 4.14 (s, 3H, NCH_3_), 2.86 (m, 2H, CH_2_C), 2.37 (s, 3H, Me_Mesityl_), 2.35 (m, 2H, CH_2_), 1.67 (s, 6H, Me_Mesityl_). ^13^C-NMR (75 MHz, CD_3_CN) δ 184.3 (C_im_Au), 170.9 (C_tz_Au), 148.3 (C_tz_), 140.4 (C_Mesityl_), 140.1 (C_Ar_), 135.6 (C_Mesityl_), 131.5 (ArH), 130.5 (ArH), 129.8 (CH_Mesityl_), 125.1 (ArH), 124.2 (CH_im_), 124.1 (C_Mesityl_), 123.0 (CH_im_), 50.7 (NCH_2_), 37.8 (NCH_3_), 30.4 (CH_2_), 22.7 (CH_2_C), 21.1 (Me_Mesityl_), 17.5 (Me_Mesityl_).

### 3.4. Crystal Structure Determination

Data were collected using an Oxford Diffraction Gemini E diffractometer, equipped with a 2K × 2K EOS CCD area detector and sealed–tube Enhance (Mo) and (Cu) X–ray sources. Single crystals of compounds have been fastened on the top of a Lindemann glass capillary. Data have been collected by means of the ω-scans technique using graphite-monochromated radiation. Detector distance has been set at 45 mm. The diffraction intensities have been corrected for Lorentz/polarization effects, as well as with respect to absorption. Empirical multi-scan absorption corrections using equivalent reflections have been performed with the scaling algorithm SCALE3 ABSPACK. Data reduction, finalization and cell refinement were carried out through the CrysAlisPro software (1.171.38.46, Rigaku Oxford Diffraction, Rigaku Corporation, Oxford, UK: 2015). Accurate unit cell parameters were obtained by least squares refinement of the angular settings of strongest reflections, chosen from the whole experiment. The structures were solved with Olex2 [46] by using ShelXT [47] structure solution program by Intrinsic Phasing and refined with the ShelXL [48] refinement package using least-squares minimization. In the last cycles of refinement, non-hydrogen atoms were refined anisotropically. Hydrogen atoms were included in calculated positions, and a riding model was used for their refinement. The specific refinement details for each of the compounds are embedded in their CIF files that have been deposited with the Cambridge Crystallographic Data Centre as supplementary publication (CCDC 1903103-1903107). Copies of the data can be obtained free of charge on application to the CCDC, 12 Union Road, Cambridge CB2 1EZ, U.K. (fax, (+44) 1223 336033; e-mail, deposit@ccdc.cam.ac.uk).

### 3.5. Luminescence Measurements

The luminescence spectra of films obtained by drop-casting from acetonitrile solutions on quartz slides were recorded at room temperature using a Fluorolog-3, Horiba JobinYvon spectrofluorimeter equipped with double-grating monochromator in both the excitation and emission sides. A 450 W Xe arc lamp and an R928P Hamamatsu photomultiplier were employed as excitation source and detector, respectively. The emission spectra were corrected for detection and optical spectral response of the spectrofluorimeter supplied by the manufacturer. 

## 4. Conclusions

In this work, different strategies for the synthesis of heterobimetallic complexes with heteroditopic *n*NHC-*tz*NHC carbene ligands were explored. The best results were obtained with a three-step procedure involving: (i) Metal coordination of an *n*NHC functionalized with a triazole pendant ring; (ii) methylation of the triazole ring; and (iii) coordination of the *tz*NHC to a second and different metal center. The Au-Ag bimetallic complex synthesized in this way can be the precursor of other bimetallic Au-M (M = transition metal center other than Au) complexes, via transmetalation reactions and we are currently exploring this reactivity. 

The approach described in this work for the synthesis of heterobimetallic complexes appears quite general, since it could be applied to a broad class of ligands, as long as the proper azolium-pendant azole precursor could be isolated [35,49,50,51]. 

## Supplementary Materials

The following are available online, Figures S1–S25: ^1^H- and ^13^C-NMR spectra of the new compounds synthesized in this work. Table S1: crystal data and structure refinement for compounds **3b**, **4**, **6**, **7** and **11**. Cif and checkcif files of the crystal structures.
