# Supplementary material for: Possible Synthetic Approaches for Heterobimetallic Complexes by Using nNHC/tzNHC Heteroditopic Carbene Ligands
Source: Molecules. 2019 Jun 21;24(12):2305. doi: 10.3390/molecules24122305 (PMC6631958; doi:10.3390/molecules24122305)

# checkCIF/PLATON report

Structure factors have been supplied for datablock(s) 4

THIS REPORT IS FOR GUIDANCE ONLY. IF USED AS PART OF A REVIEW PROCEDURE FOR PUBLICATION, IT SHOULD NOT REPLACE THE EXPERTISE OF AN EXPERIENCED CRYSTALLOGRAPHIC REFEREE.

No syntax errors found. CIF dictionary Interpreting this report

# Datablock: 4

---

```
Bond precision:  C-C = 0.0126 A          Wavelength=1.54184

Cell:            a=6.5131(3)    b=13.8875(5)        c=11.8617(5)
                 alpha=90       beta=103.920(4)     gamma=90
Temperature:     297 K

                Calculated                Reported
Volume          1041.39(8)                1041.39(8)
Space group     P 21/n                    P 1 21/n 1
Hall group      -P 2yn                    -P 2yn
Moiety formula  C18 H24 Au N4, I          C18 H24 Au N4, I
Sum formula     C18 H24 Au I N4           C18 H24 Au I N4
Mr              620.28                    620.28
Dx,g cm-3       1.978                     1.978
Z               2                         2
Mu (mm-1)       24.952                    24.952
F000            584.0                     584.0
F000'           575.97
h,k,lmax        7,15,13                   7,15,13
Nref            1497                      1492
Tmin,Tmax       0.137,0.136               0.021,1.000
Tmin'           0.005

Correction method= # Reported T Limits: Tmin=0.021 Tmax=1.000
AbsCorr = MULTI-SCAN

Data completeness= 0.997          Theta(max)= 58.933

R(reflections)= 0.0496( 1205)     wR2(reflections)= 0.1532( 1492)

S = 1.020                 Npar= 113
```

---

The following ALERTS were generated. Each ALERT has the format
**test-name_ALERT_alert-type_alert-level**.
Click on the hyperlinks for more details of the test.

### Alert level B

```
THETM01_ALERT_3_B  The value of sine(theta_max)/wavelength is less than 0.575
            Calculated sin(theta_max)/wavelength =    0.5555
PLAT023_ALERT_3_B Resolution (too) Low [sin(theta)/Lambda < 0.6]..      58.93 Degree
```

### Alert level C

```
PLAT342_ALERT_3_C Low Bond Precision on  C-C Bonds ...............     0.0126 Ang.
PLAT911_ALERT_3_C Missing FCF Refl Between Thmin & STh/L=    0.556          5 Report
PLAT972_ALERT_2_C Check Calcd Resid. Dens.  0.77A   From Au1            -1.69 eA-3
PLAT972_ALERT_2_C Check Calcd Resid. Dens.  0.31A   From I1             -1.60 eA-3
PLAT977_ALERT_2_C Check Negative Difference Density on H5B              -0.58 eA-3
PLAT978_ALERT_2_C Number C-C Bonds with Positive Residual Density.          0 Info
```

### Alert level G

```
PLAT012_ALERT_1_G No       _shelx_res_checksum Found in CIF ......     Please Check
PLAT072_ALERT_2_G SHELXL First  Parameter in WGHT  Unusually Large       0.11 Report
PLAT304_ALERT_4_G Non-Integer Number of Atoms in ...... Resd  2          0.50 Check
PLAT909_ALERT_3_G Percentage of I>2sig(I) Data at Theta(Max) Still        76% Note
PLAT933_ALERT_2_G Number of OMIT Records in Embedded .res File ...          5 Note
```

```
   0 ALERT level A = Most likely a serious problem - resolve or explain
   2 ALERT level B = A potentially serious problem, consider carefully
   6 ALERT level C = Check. Ensure it is not caused by an omission or oversight
   5 ALERT level G = General information/check it is not something unexpected

   1 ALERT type 1 CIF construction/syntax error, inconsistent or missing data
   6 ALERT type 2 Indicator that the structure model may be wrong or deficient
   5 ALERT type 3 Indicator that the structure quality may be low
   1 ALERT type 4 Improvement, methodology, query or suggestion
   0 ALERT type 5 Informative message, check
```

## checkCIF publication errors

### Alert level A

```
PUBL004_ALERT_1_A  The contact author's name and address are missing,
            _publ_contact_author_name and _publ_contact_author_address.
PUBL005_ALERT_1_A  _publ_contact_author_email, _publ_contact_author_fax and
            _publ_contact_author_phone are all missing.
            At least one of these should be present.
PUBL006_ALERT_1_A  _publ_requested_journal is missing
            e.g. 'Acta Crystallographica Section C'
PUBL008_ALERT_1_A  _publ_section_title is missing. Title of paper.
PUBL009_ALERT_1_A  _publ_author_name is missing. List of author(s) name(s).
PUBL010_ALERT_1_A  _publ_author_address is missing. Author(s) address(es).
PUBL012_ALERT_1_A  _publ_section_abstract is missing.
            Abstract of paper in English.
```

```
   7 ALERT level A = Data missing that is essential or data in wrong format
   0 ALERT level G = General alerts. Data that may be required is missing
```

**Publication of your CIF**

You should attempt to resolve as many as possible of the alerts in all categories. Often the minor alerts point to easily fixed oversights, errors and omissions in your CIF or refinement strategy, so attention to these fine details can be worthwhile. In order to resolve some of the more serious problems it may be necessary to carry out additional measurements or structure refinements. However, the nature of your study may justify the reported deviations from journal submission requirements and the more serious of these should be commented upon in the discussion or experimental section of a paper or in the "special_details" fields of the CIF. *checkCIF* was carefully designed to identify outliers and unusual parameters, but every test has its limitations and alerts that are not important in a particular case may appear. Conversely, the absence of alerts does not guarantee there are no aspects of the results needing attention. It is up to the individual to critically assess their own results and, if necessary, seek expert advice.

If level A alerts remain, which you believe to be justified deviations, and you intend to submit this CIF for publication in a journal, you should additionally insert an explanation in your CIF using the Validation Reply Form (VRF) below. This will allow your explanation to be considered as part of the review process.

**Validation response form**

Please find below a validation response form (VRF) that can be filled in and pasted into your CIF.

```
# start Validation Reply Form
_vrf_PUBL004_GLOBAL
;
PROBLEM: The contact author’s name and address are missing,
RESPONSE: ...
;
_vrf_PUBL005_GLOBAL
;
PROBLEM: _publ_contact_author_email, _publ_contact_author_fax and
RESPONSE: ...
;
_vrf_PUBL006_GLOBAL
;
PROBLEM: _publ_requested_journal is missing
RESPONSE: ...
;
_vrf_PUBL008_GLOBAL
;
PROBLEM: _publ_section_title is missing. Title of paper.
RESPONSE: ...
;
_vrf_PUBL009_GLOBAL
;
PROBLEM: _publ_author_name is missing. List of author(s) name(s).
RESPONSE: ...
;
_vrf_PUBL010_GLOBAL
;
PROBLEM: _publ_author_address is missing. Author(s) address(es).
RESPONSE: ...
;
_vrf_PUBL012_GLOBAL
;
```

```
PROBLEM: _publ_section_abstract is missing.
RESPONSE: ...
;
# end Validation Reply Form
```

If you wish to submit your CIF for publication in Acta Crystallographica Section C or E, you should upload your CIF via the web. If you wish to submit your CIF for publication in IUCrData you should upload your CIF via the web. If your CIF is to form part of a submission to another IUCr journal, you will be asked, either during electronic submission or by the Co-editor handling your paper, to upload your CIF via our web site.

---

**PLATON version of 18/02/2019; check.def file version of 18/02/2019**

**Datablock 4** - ellipsoid plot

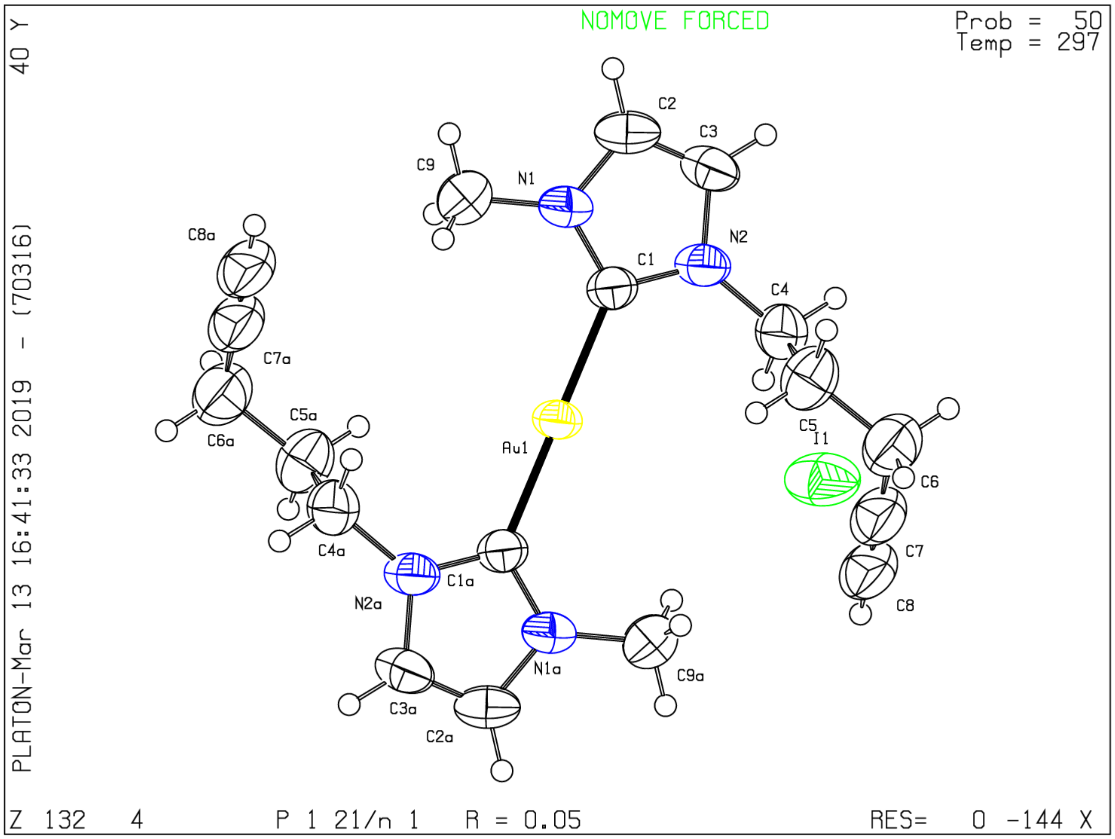

Supplement: Supplementary file 1 [file molecules-24-02305-s001.zip › 4_cifreport.pdf]
