# Supplementary material for: Possible Synthetic Approaches for Heterobimetallic Complexes by Using nNHC/tzNHC Heteroditopic Carbene Ligands
Source: Molecules. 2019 Jun 21;24(12):2305. doi: 10.3390/molecules24122305 (PMC6631958; doi:10.3390/molecules24122305)

checkCIF/PLATON report

Structure factors have been supplied for datablock(s) 7

THIS REPORT IS FOR GUIDANCE ONLY. IF USED AS PART OF A REVIEW PROCEDURE FOR PUBLICATION, IT SHOULD NOT REPLACE THE EXPERTISE OF AN EXPERIENCED CRYSTALLOGRAPHIC REFEREE.

No syntax errors found.   CIF dictionary   Interpreting this report

Datablock: 7

```
Bond precision:  C-C = 0.0267 A          Wavelength=1.54184

Cell:           a=9.3633(2)         b=14.5000(4)     c=23.1244(7)
                alpha=106.078(2)    beta=91.868(2)   gamma=108.675(2)
Temperature:    298 K
```

	Calculated	Reported
Volume	2832.44(14)	2832.44(14)
Space group	P -1	P -1
Hall group	-P 1	-P 1
Moiety formula	3(C30 H34 Au I2 N10), 2(C2 H3 N), 3(I)	3(C30 H34 Au I2 N10), 3(I), 2(C2 H3 N)
Sum formula	C94 H108 Au3 I9 N32	C94 H108 Au3 I9 N32
Mr	3419.13	3419.12
Dx,g cm-3	2.004	2.004
Z	1	1
Mu (mm-1)	26.869	26.869
F000	1610.0	1610.0
F000’	1598.38	
h,k,lmax	11,17,27	11,17,27
Nref	9624	9336
Tmin,Tmax	0.076,0.068	0.065,1.000
Tmin’	0.002	

```
Correction method= # Reported T Limits: Tmin=0.065 Tmax=1.000
AbsCorr = MULTI-SCAN

Data completeness= 0.970          Theta(max)= 65.078

R(reflections)= 0.0594( 8132)     wR2(reflections)= 0.1553( 9336)

S = 1.062                   Npar= 629
```

```
The following ALERTS were generated. Each ALERT has the format
       test-name_ALERT_alert-type_alert-level.
Click on the hyperlinks for more details of the test.
```

Alert level A

```
PLAT971_ALERT_2_A Check Calcd Resid. Dens.  0.93A   From Au1            3.51 eA-3
```

Author Response: The large residual density is close to the an heavy atom. It may be caused by the inappropriate atomic scattering factor and the absorption correction.

Alert level B

```
PLAT342_ALERT_3_B Low Bond Precision on  C-C Bonds ...............    0.02668 Ang.
PLAT434_ALERT_2_B Short Inter HL..HL Contact I2        ..I5              3.42 Ang.
                                                     x,y,z  =      1_555 Check
```

Alert level C

```
THETM01_ALERT_3_C  The value of sine(theta_max)/wavelength is less than 0.590
            Calculated sin(theta_max)/wavelength =    0.5882
PLAT029_ALERT_3_C _diffrn_measured_fraction_theta_full value Low .      0.970 Why?
PLAT220_ALERT_2_C Non-Solvent  Resd 1  C   Ueq(max)/Ueq(min) Range        4.8 Ratio
PLAT241_ALERT_2_C High   'MainMol' Ueq as Compared to Neighbors of         N8 Check
PLAT241_ALERT_2_C High   'MainMol' Ueq as Compared to Neighbors of        C16 Check
PLAT242_ALERT_2_C Low    'MainMol' Ueq as Compared to Neighbors of        C12 Check
PLAT242_ALERT_2_C Low    'MainMol' Ueq as Compared to Neighbors of        C14 Check
PLAT244_ALERT_4_C Low    'Solvent' Ueq as Compared to Neighbors of        C46 Check
PLAT250_ALERT_2_C Large U3/U1 Ratio for Average U(i,j) Tensor ....        2.3 Note
PLAT260_ALERT_2_C Large Average Ueq of Residue Including       N16      0.138 Check
PLAT331_ALERT_2_C Small Aver Phenyl C-C Dist C25       -C30      .       1.37 Ang.
PLAT906_ALERT_3_C Large K Value in the Analysis of Variance ......      4.298 Check
PLAT911_ALERT_3_C Missing FCF Refl Between Thmin & STh/L=    0.588        287 Report
PLAT971_ALERT_2_C Check Calcd Resid. Dens.  0.96A   From I1             2.34 eA-3
```

Author Response: The large residual density is close to the an heavy atom. It may be caused by the inappropriate atomic scattering factor and the absorption correction.

```
PLAT971_ALERT_2_C Check Calcd Resid. Dens.  0.86A   From Au1            2.17 eA-3
```

Author Response: The large residual density is close to the an heavy atom. It may be caused by the inappropriate atomic scattering factor and the absorption correction.

```
PLAT971_ALERT_2_C Check Calcd Resid. Dens.  0.97A   From Au2            1.90 eA-3
```

Author Response: The large residual density is close to the an heavy atom. It may be caused by the inappropriate atomic scattering factor and the absorption correction.

```
PLAT971_ALERT_2_C Check Calcd Resid. Dens.  1.31A   From Au2            1.71 eA-3
```

Author Response: The large residual density is close to the an heavy atom. It may be caused by the inappropriate atomic scattering factor and the absorption correction.

```
PLAT971_ALERT_2_C Check Calcd Resid. Dens.  0.88A   From I1             1.59 eA-3
```

Author Response: The large residual density is close to the an heavy atom. It may be caused by the inappropriate atomic scattering factor and the absorption correction.

```
PLAT971_ALERT_2_C Check Calcd Resid. Dens.  0.92A   From I4             1.57 eA-3
```

Author Response: The large residual density is close to the an heavy atom. It may be caused by the inappropriate atomic scattering factor and the absorption correction.

```
PLAT971_ALERT_2_C Check Calcd Resid. Dens.  0.84A   From I3             1.55 eA-3
```

Author Response: The large residual density is close to the an heavy atom. It may be caused by the inappropriate atomic scattering factor and the absorption correction.

```
PLAT971_ALERT_2_C Check Calcd Resid. Dens.  0.95A   From I4             1.54 eA-3
```

Author Response: The large residual density is close to the an heavy atom. It may be caused by the inappropriate atomic scattering factor and the absorption correction.

```
PLAT971_ALERT_2_C Check Calcd Resid. Dens.  0.84A   From I3             1.54 eA-3
```

Author Response: The large residual density is close to the an heavy atom. It may be caused by the inappropriate atomic scattering factor and the absorption correction.

```
PLAT972_ALERT_2_C Check Calcd Resid. Dens.  0.78A   From Au1           -2.15 eA-3
PLAT972_ALERT_2_C Check Calcd Resid. Dens.  0.65A   From Au1           -1.64 eA-3
PLAT972_ALERT_2_C Check Calcd Resid. Dens.  1.57A   From Au1           -1.60 eA-3
PLAT972_ALERT_2_C Check Calcd Resid. Dens.  1.22A   From Au2           -1.56 eA-3
PLAT972_ALERT_2_C Check Calcd Resid. Dens.  0.85A   From I1            -1.55 eA-3
PLAT972_ALERT_2_C Check Calcd Resid. Dens.  0.72A   From Au1           -1.54 eA-3
PLAT972_ALERT_2_C Check Calcd Resid. Dens.  1.13A   From C14           -1.53 eA-3
PLAT977_ALERT_2_C Check Negative Difference Density on H21B            -0.36 eA-3
PLAT977_ALERT_2_C Check Negative Difference Density on H24             -0.45 eA-3
PLAT977_ALERT_2_C Check Negative Difference Density on H26             -0.33 eA-3
PLAT977_ALERT_2_C Check Negative Difference Density on H34B            -0.46 eA-3
PLAT977_ALERT_2_C Check Negative Difference Density on H39             -0.31 eA-3
PLAT977_ALERT_2_C Check Negative Difference Density on H43             -0.39 eA-3
PLAT978_ALERT_2_C Number C-C Bonds with Positive Residual Density.         0 Info
```

Alert level G

```
PLAT003_ALERT_2_G Number of Uiso or Uij Restrained non-H Atoms ...         70 Report
PLAT012_ALERT_1_G No       _shelx_res_checksum Found in CIF ......     Please Check
PLAT042_ALERT_1_G Calc. and Reported MoietyFormula Strings  Differ     Please Check
PLAT083_ALERT_2_G SHELXL Second Parameter in WGHT  Unusually Large      43.62 Why ?
PLAT154_ALERT_1_G The s.u.'s on the Cell Angles are Equal ..(Note)      0.002 Degree
PLAT186_ALERT_4_G The CIF-Embedded .res File Contains ISOR Records          1 Report
PLAT187_ALERT_4_G The CIF-Embedded .res File Contains RIGU Records          1 Report
PLAT304_ALERT_4_G Non-Integer Number of Atoms in ...... Resd  5          0.50 Check
PLAT721_ALERT_1_G Bond    Calc     0.97000, Rep     0.96000 Dev...       0.01 Ang.
              C47      -H47C            1.555   1.555 ........     #  130 Check
PLAT790_ALERT_4_G Centre of Gravity not Within Unit Cell: Resd.  #          3 Note
              C2 H3 N
PLAT860_ALERT_3_G Number of Least-Squares Restraints ............         520 Note
PLAT909_ALERT_3_G Percentage of I>2sig(I) Data at Theta(Max) Still        77% Note
PLAT910_ALERT_3_G Missing # of FCF Reflection(s) Below Theta(Min).          1 Note
PLAT933_ALERT_2_G Number of OMIT Records in Embedded .res File ...         47 Note
```

---

1 **ALERT level A** = Most likely a serious problem - resolve or explain
2 **ALERT level B** = A potentially serious problem, consider carefully
36 **ALERT level C** = Check. Ensure it is not caused by an omission or oversight
14 **ALERT level G** = General information/check it is not something unexpected

4 ALERT type 1 CIF construction/syntax error, inconsistent or missing data
36 ALERT type 2 Indicator that the structure model may be wrong or deficient
8 ALERT type 3 Indicator that the structure quality may be low
5 ALERT type 4 Improvement, methodology, query or suggestion
0 ALERT type 5 Informative message, check

---

checkCIF publication errors

Alert level A

```
PUBL004_ALERT_1_A  The contact author's name and address are missing,
            _publ_contact_author_name and _publ_contact_author_address.
PUBL005_ALERT_1_A  _publ_contact_author_email, _publ_contact_author_fax and
            _publ_contact_author_phone are all missing.
            At least one of these should be present.
PUBL006_ALERT_1_A  _publ_requested_journal is missing
            e.g. 'Acta Crystallographica Section C'
PUBL008_ALERT_1_A  _publ_section_title is missing. Title of paper.
PUBL009_ALERT_1_A  _publ_author_name is missing. List of author(s) name(s).
PUBL010_ALERT_1_A  _publ_author_address is missing. Author(s) address(es).
PUBL012_ALERT_1_A  _publ_section_abstract is missing.
            Abstract of paper in English.
```

---

7 **ALERT level A** = Data missing that is essential or data in wrong format
0 **ALERT level G** = General alerts. Data that may be required is missing

---

Publication of your CIF

You should attempt to resolve as many as possible of the alerts in all categories. Often the minor alerts point to easily fixed oversights, errors and omissions in your CIF or refinement strategy, so attention to these fine details can be worthwhile. In order to resolve some of the more serious problems it may be necessary to carry out additional measurements or structure refinements. However, the nature of your study may justify the reported deviations from journal submission requirements and the more serious of these should be commented upon in the discussion or experimental section of a paper or in the "special_details" fields of the CIF. *checkCIF* was carefully designed to identify outliers and unusual parameters, but every test has its limitations and alerts that are not important in a particular case may appear. Conversely, the absence of alerts does not guarantee there are no aspects of the results needing attention. It is up to the individual to critically assess their own results and, if necessary, seek expert advice.

If level A alerts remain, which you believe to be justified deviations, and you intend to submit this CIF for publication in a journal, you should additionally insert an explanation in your CIF using the Validation Reply Form (VRF) below. This will allow your explanation to be considered as part of the review process.

Validation response form

Please find below a validation response form (VRF) that can be filled in and pasted into your CIF.

```
# start Validation Reply Form
_vrf_PUBL004_GLOBAL
;
PROBLEM: The contact author’s name and address are missing,
RESPONSE: ...
;
_vrf_PUBL005_GLOBAL
;
PROBLEM: _publ_contact_author_email, _publ_contact_author_fax and
RESPONSE: ...
;
_vrf_PUBL006_GLOBAL
;
PROBLEM: _publ_requested_journal is missing
RESPONSE: ...
;
_vrf_PUBL008_GLOBAL
;
PROBLEM: _publ_section_title is missing. Title of paper.
RESPONSE: ...
;
_vrf_PUBL009_GLOBAL
;
PROBLEM: _publ_author_name is missing. List of author(s) name(s).
RESPONSE: ...
;
_vrf_PUBL010_GLOBAL
;
PROBLEM: _publ_author_address is missing. Author(s) address(es).
RESPONSE: ...
;
_vrf_PUBL012_GLOBAL
;
```

```
PROBLEM: _publ_section_abstract is missing.
RESPONSE: ...
;
# end Validation Reply Form
```

If you wish to submit your CIF for publication in Acta Crystallographica Section C or E, you should upload your CIF via the web. If you wish to submit your CIF for publication in IUCrData you should upload your CIF via the web. If your CIF is to form part of a submission to another IUCr journal, you will be asked, either during electronic submission or by the Co-editor handling your paper, to upload your CIF via our web site.

PLATON version of 18/02/2019; check.def file version of 18/02/2019

Datablock 7 - ellipsoid plot

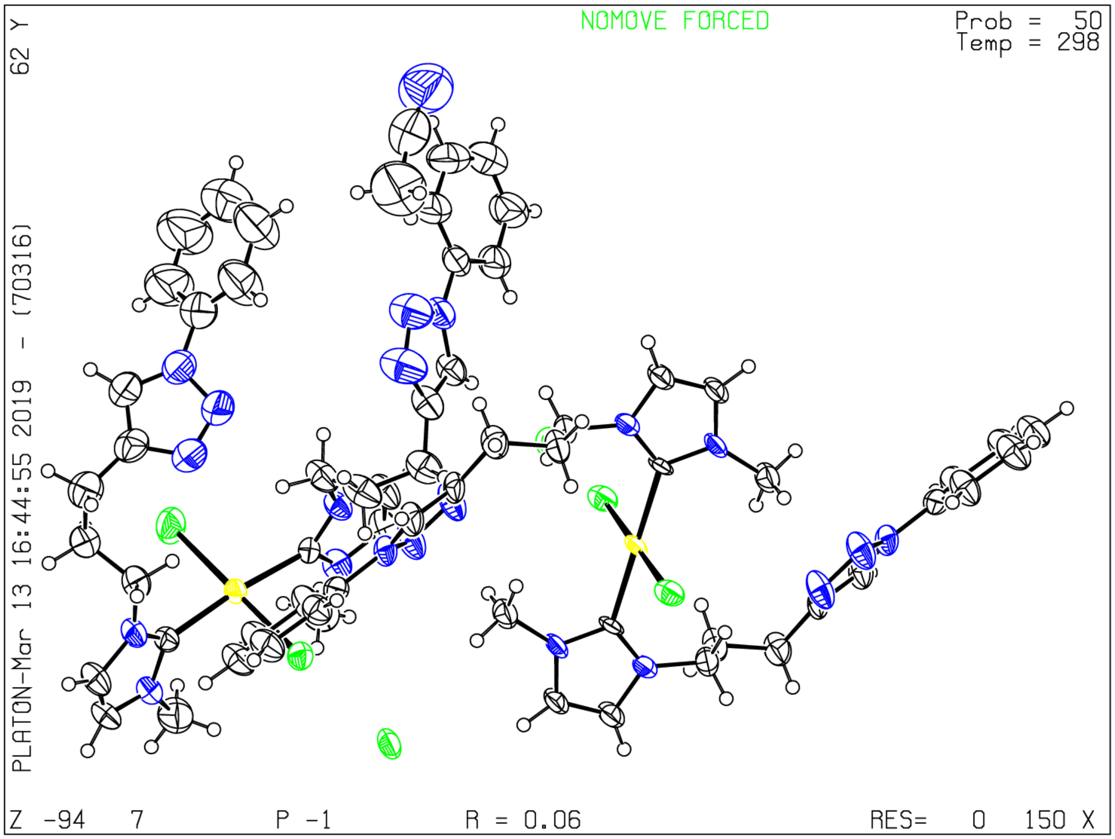

Supplement: Supplementary file 1 [file molecules-24-02305-s001.zip › 7_cifreport.pdf]
