# Supplementary material for: Possible Synthetic Approaches for Heterobimetallic Complexes by Using nNHC/tzNHC Heteroditopic Carbene Ligands
Source: Molecules. 2019 Jun 21;24(12):2305. doi: 10.3390/molecules24122305 (PMC6631958; doi:10.3390/molecules24122305)
Supplement: Supplementary file 1 [file molecules-24-02305-s001.zip › ESI_20_06.pdf]

SUPPORTING INFORMATION FOR

Possible synthetic approaches for heterobimetallic complexes by using *n*NHC/*tz*NHC heteroditopic carbene ligands

Andrea Longhi [1], Marco Baron [1], Marzio Rancan [2], Gregorio Bottaro [2], Lidia Armelao [1,2], Paolo Sgarbossa [3] and Cristina Tubaro *[1]

[1] Department of Chemical Sciences, University of Padova, via F. Marzolo 1, 35131 Padova, Italy.

[2] Institute of Condensed Matter Chemistry and Technologies for Energy, National Research Council, ICMATE-CNR, c/o Department of Chemical Sciences, University of Padova, via F. Marzolo 1, 35131 Padova, Italy.

[3] Department of Industrial Engineering, University of Padova, via F. Marzolo 9, 35131 Padova, Italy.

Table of contents:

Figures S1-S25. NMR spectra of the proligands and complexes 2

Table S1. Crystal data and structure refinement for compounds **3b**, **4**, **6**, **7** and **11**........15

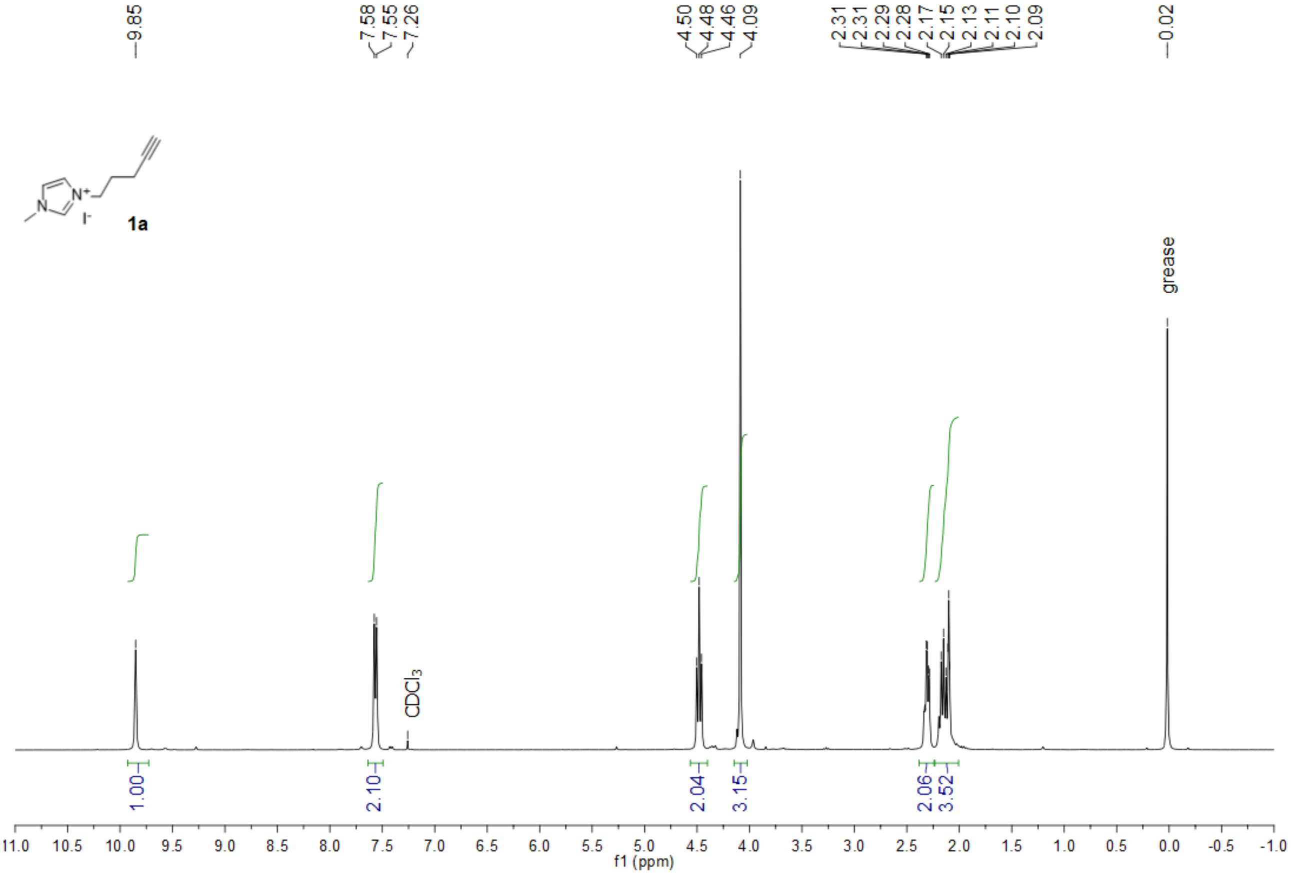

Figure S1. ^{1}H NMR of compound **1a** in $CDCl_3$.

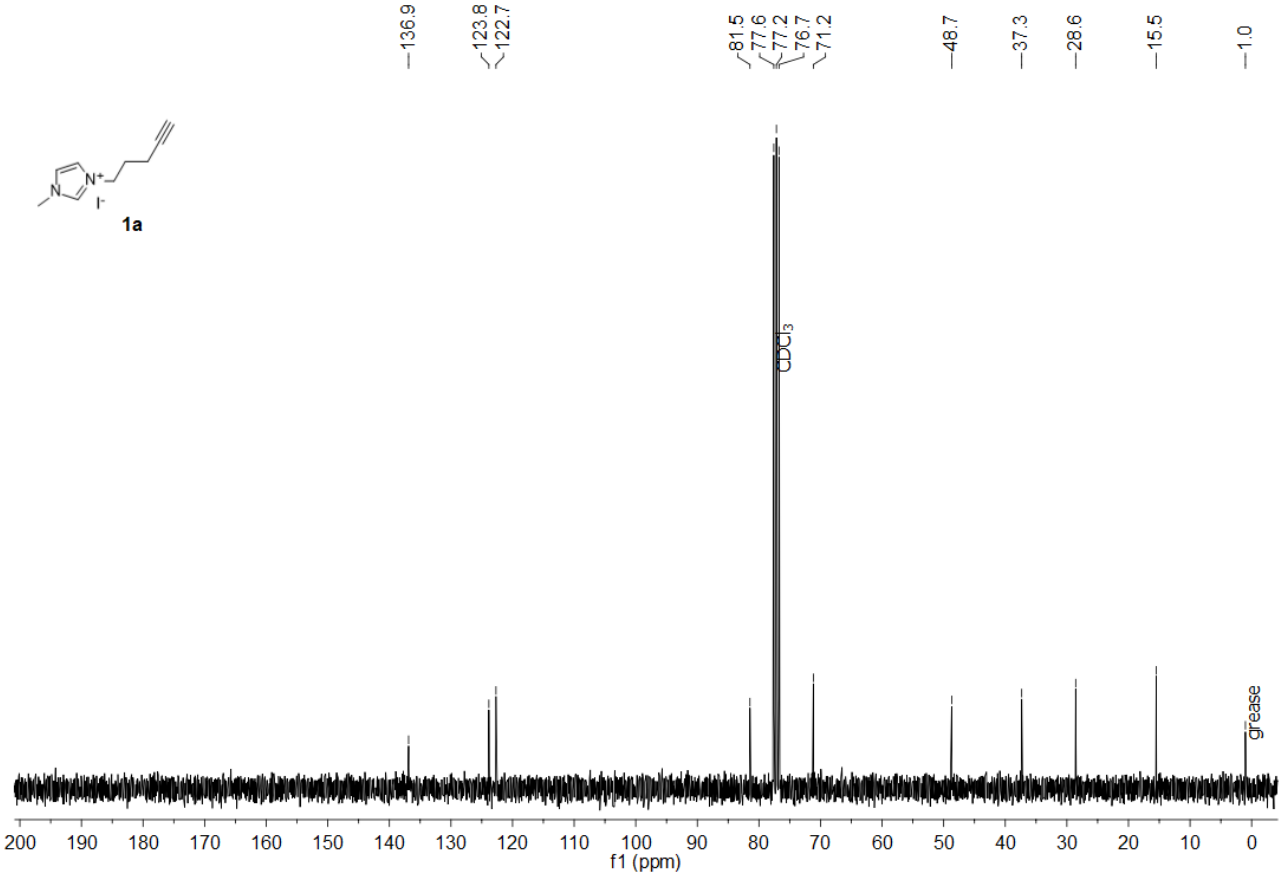

Figure S2. $^{13}C\{^{1}H\}$ NMR of compound **1a** in $CDCl_3$.

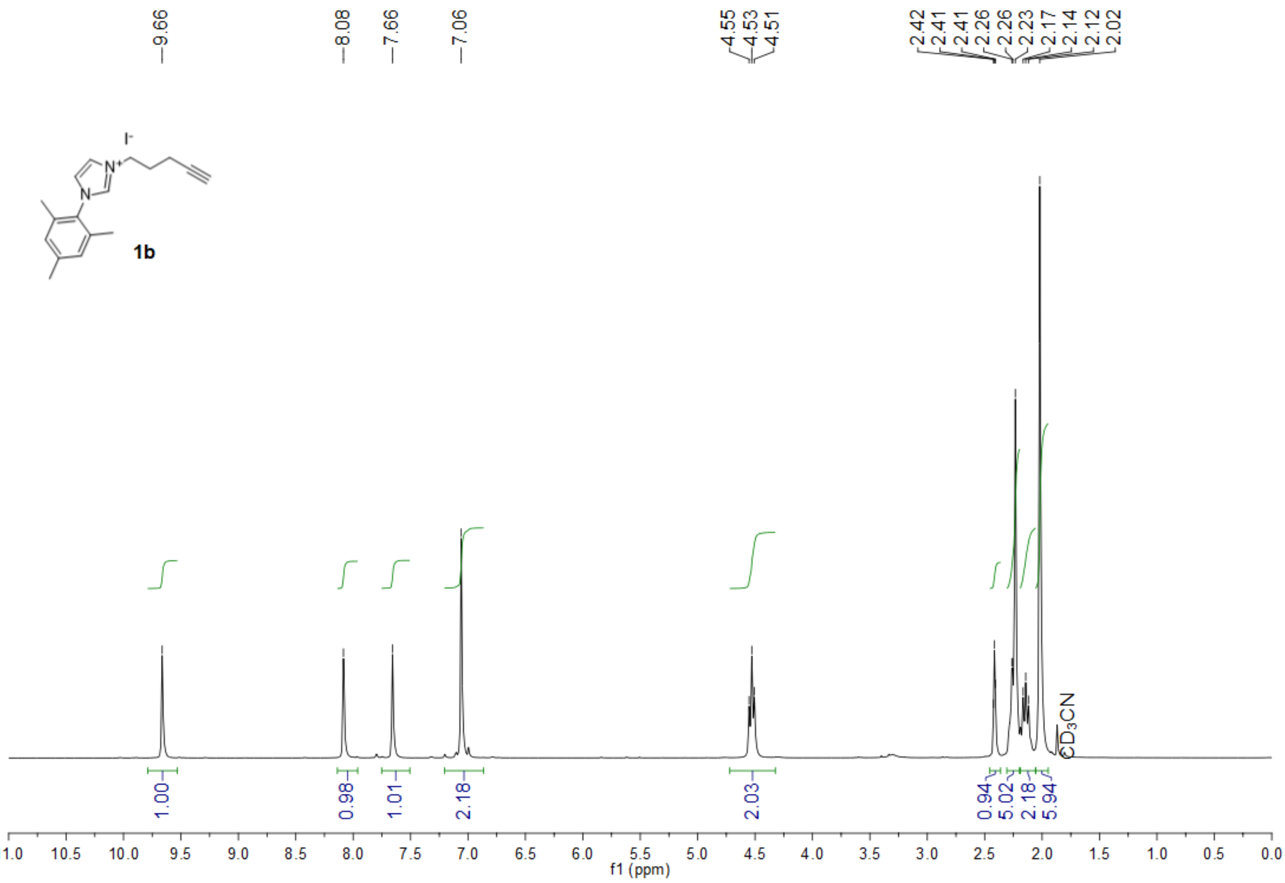

Figure S3. ^{1}H NMR of compound **1b** in CD_3CN.

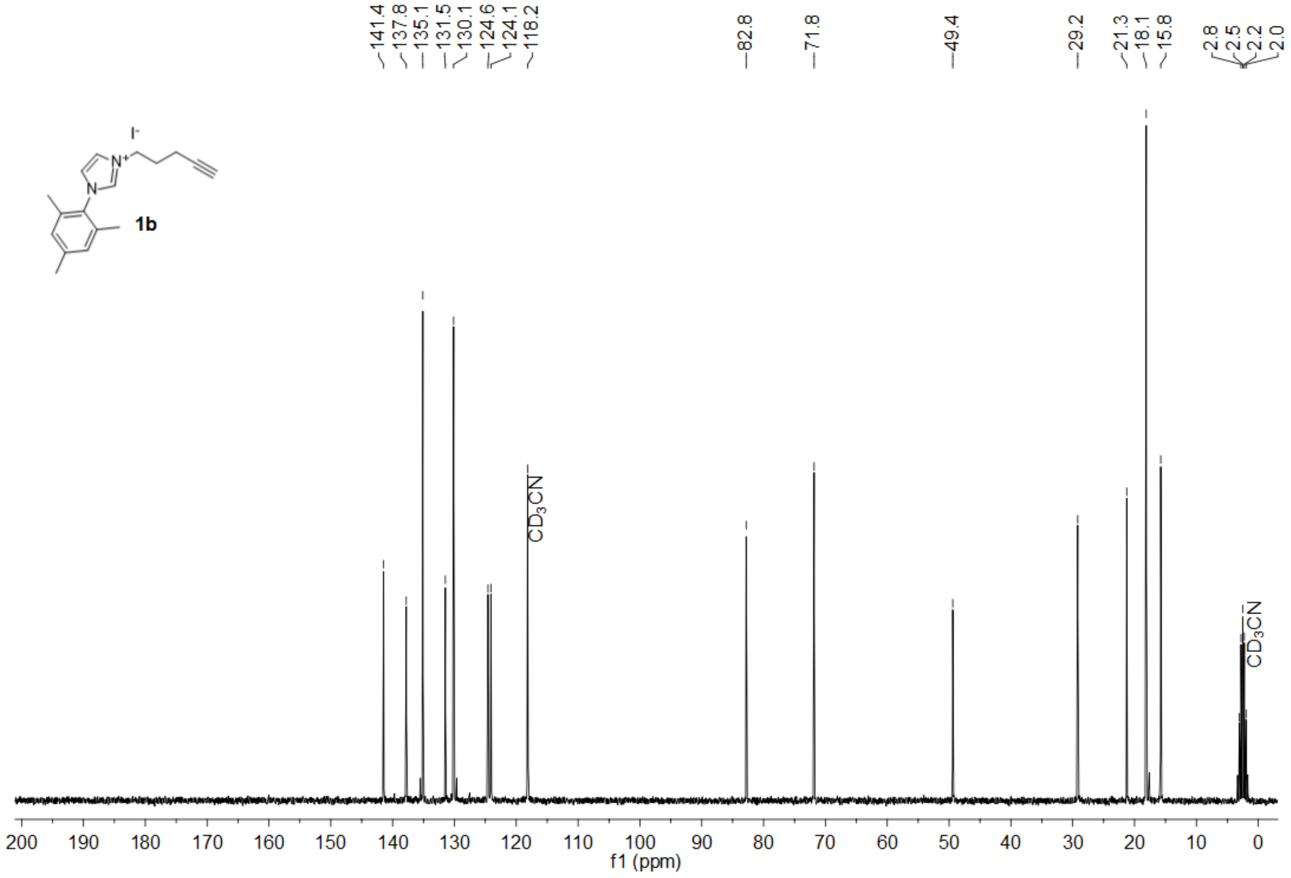

Figure S4. ^{13}C{^{1}H} NMR of compound **1b** in CD_3CN.

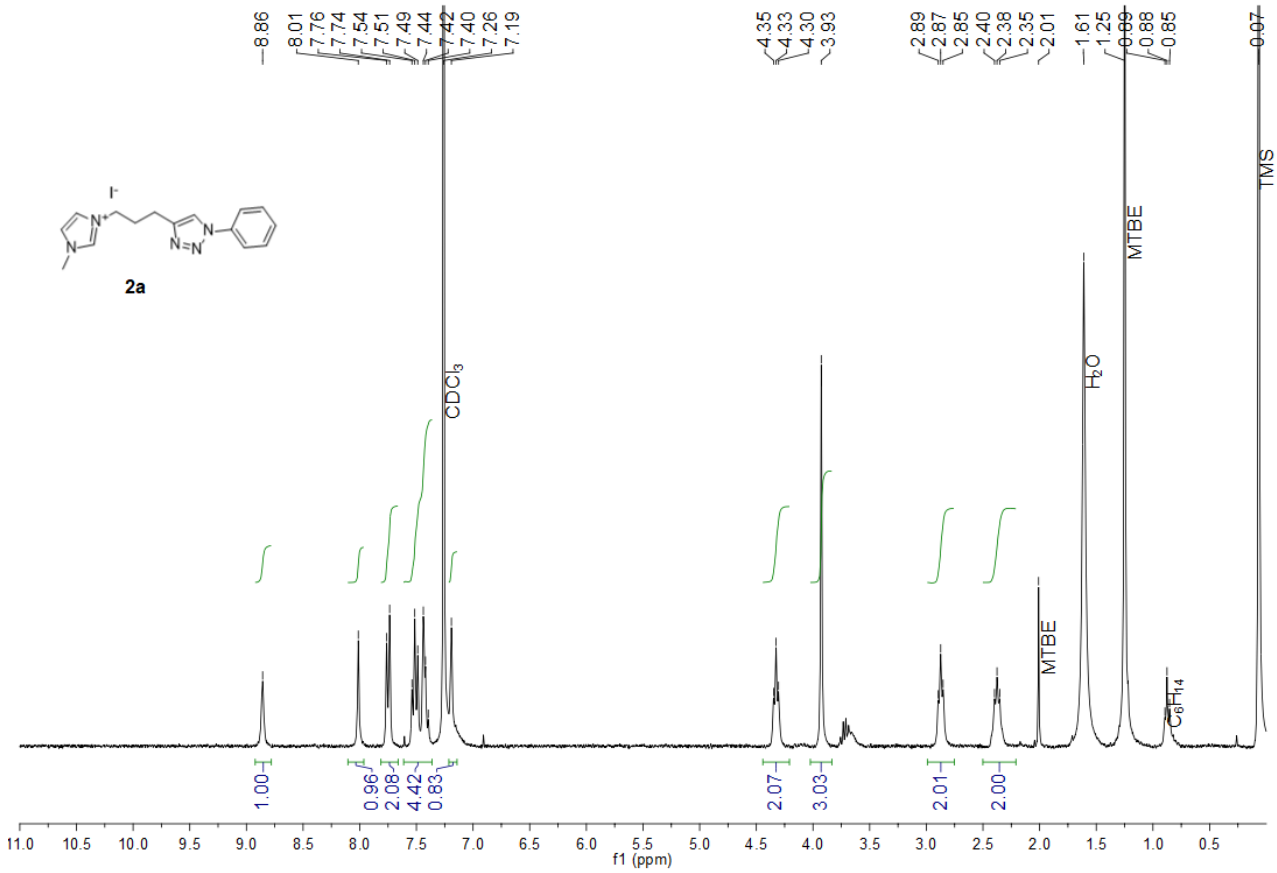

Figure S5. ^{1}H NMR of compound **2a** in $CDCl_3$.

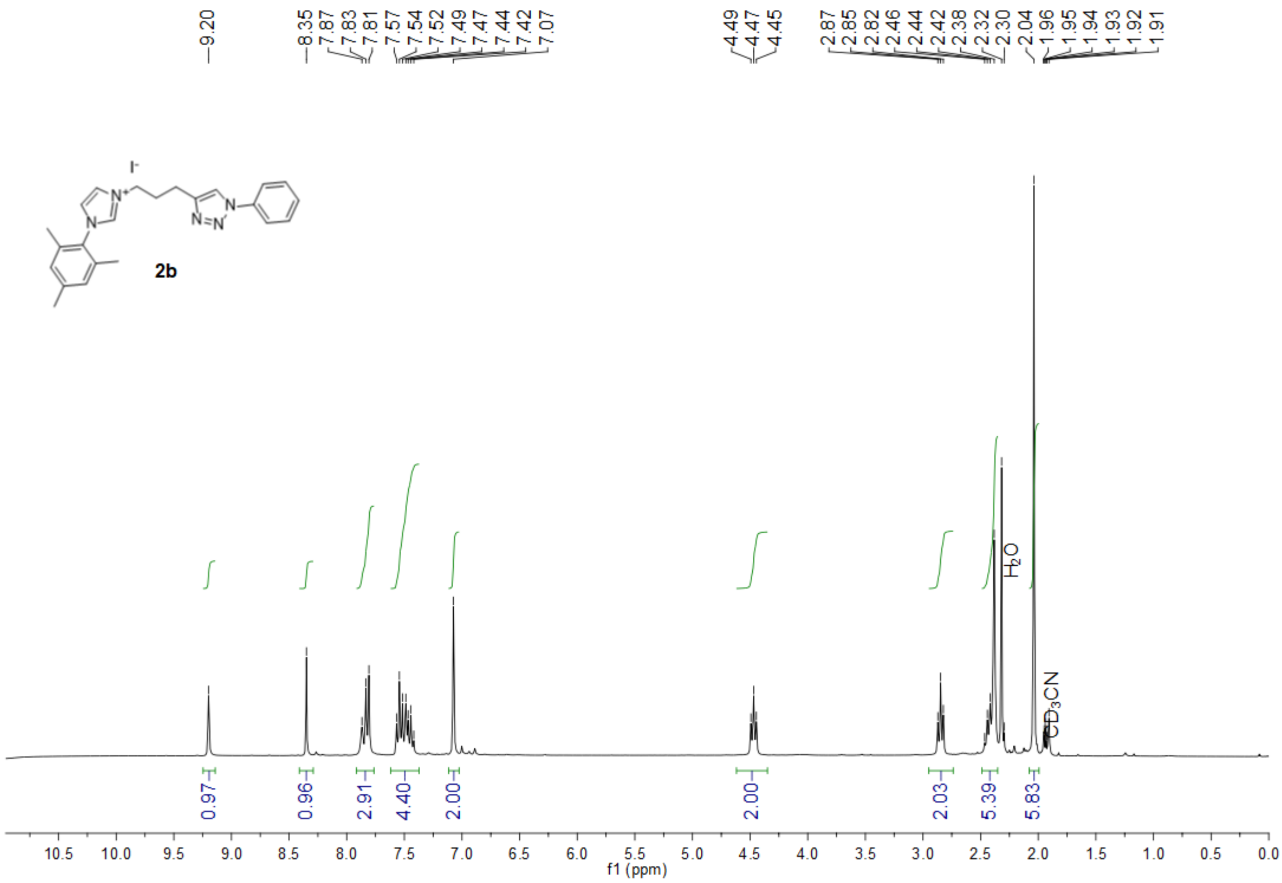

Figure S6. ^{1}H NMR of compound **2b** in CD_3CN.

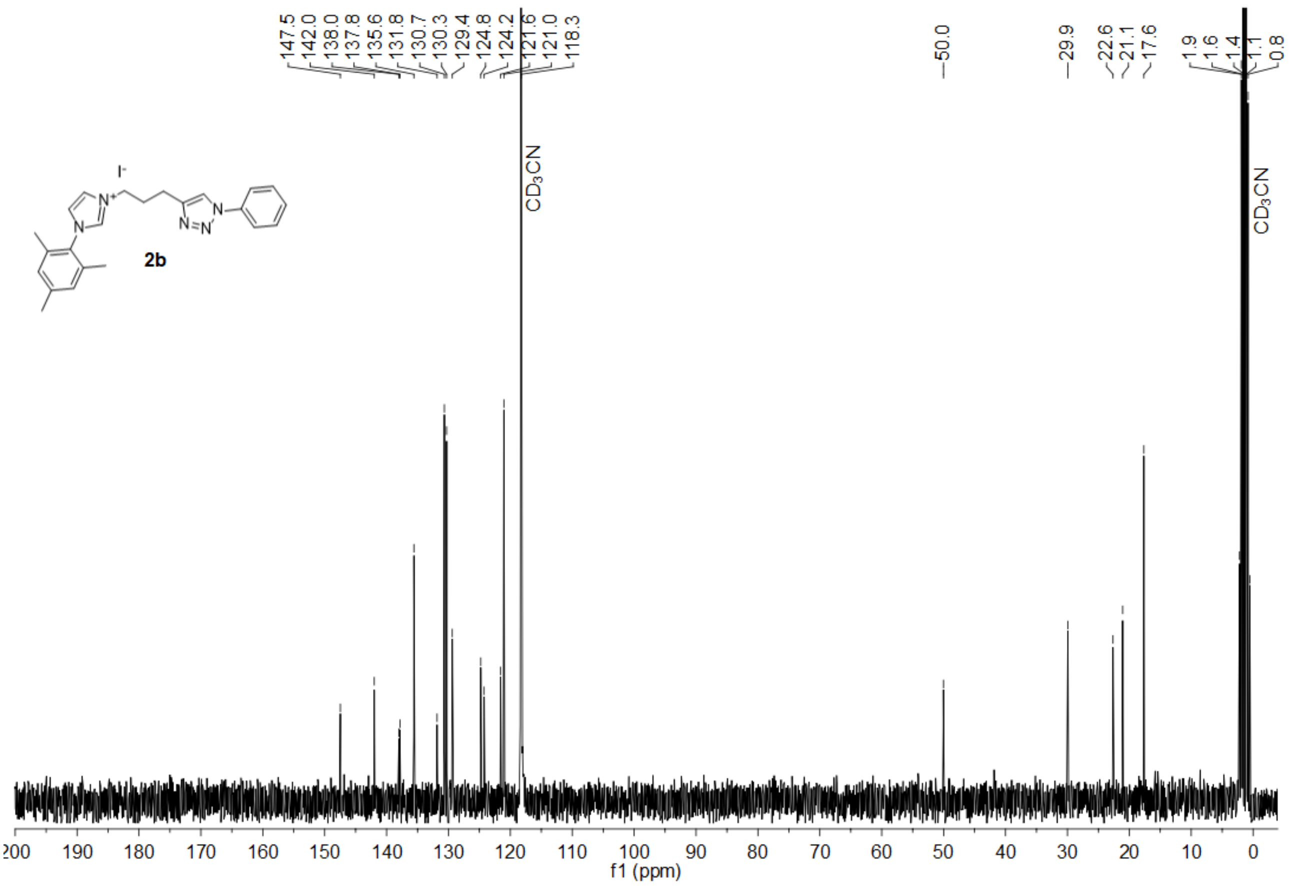

Figure S7. $^{13}C\{^{1}H\}$ NMR of compound **2b** in CD_3CN.

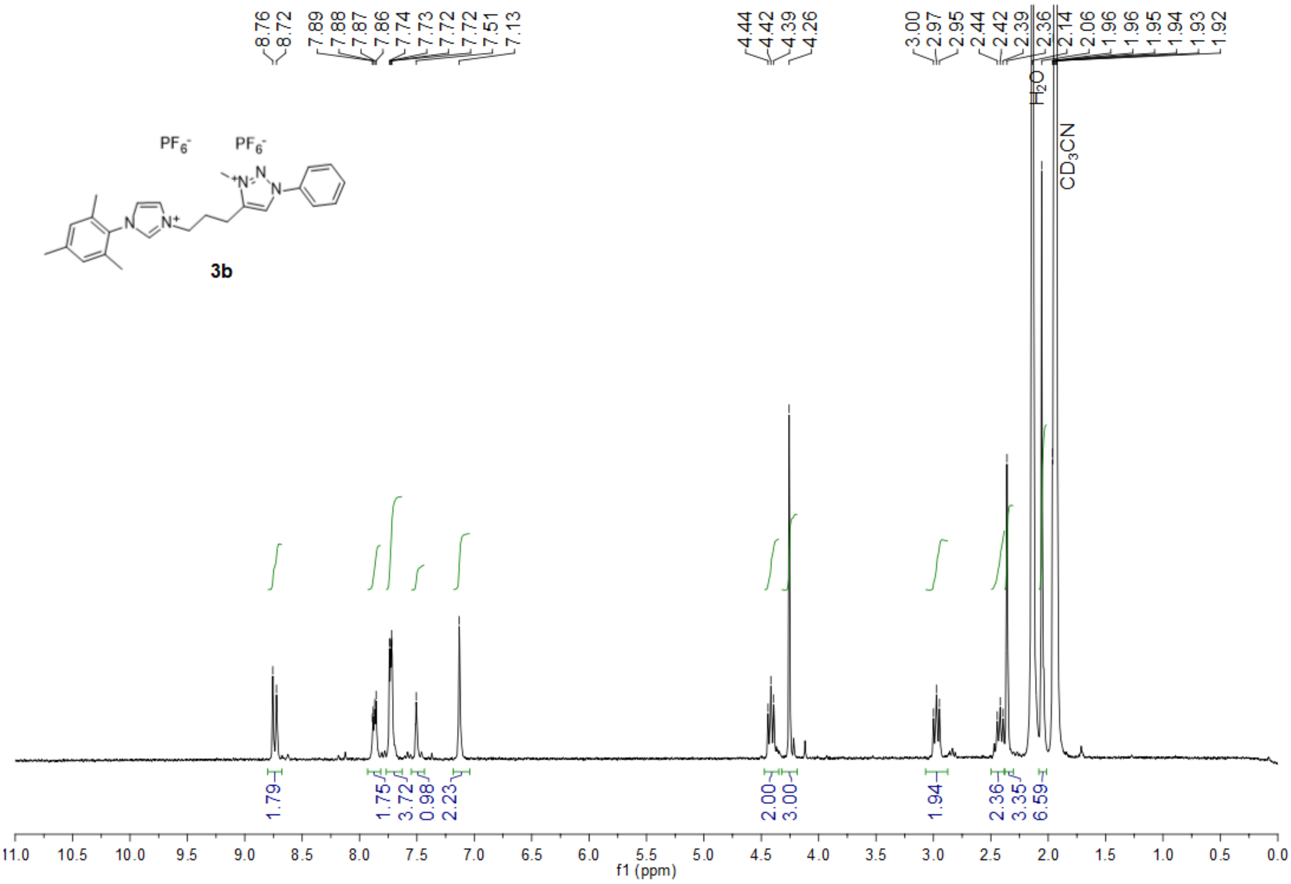

Figure S8. ^{1}H NMR of compound **3b** in CD_3CN.

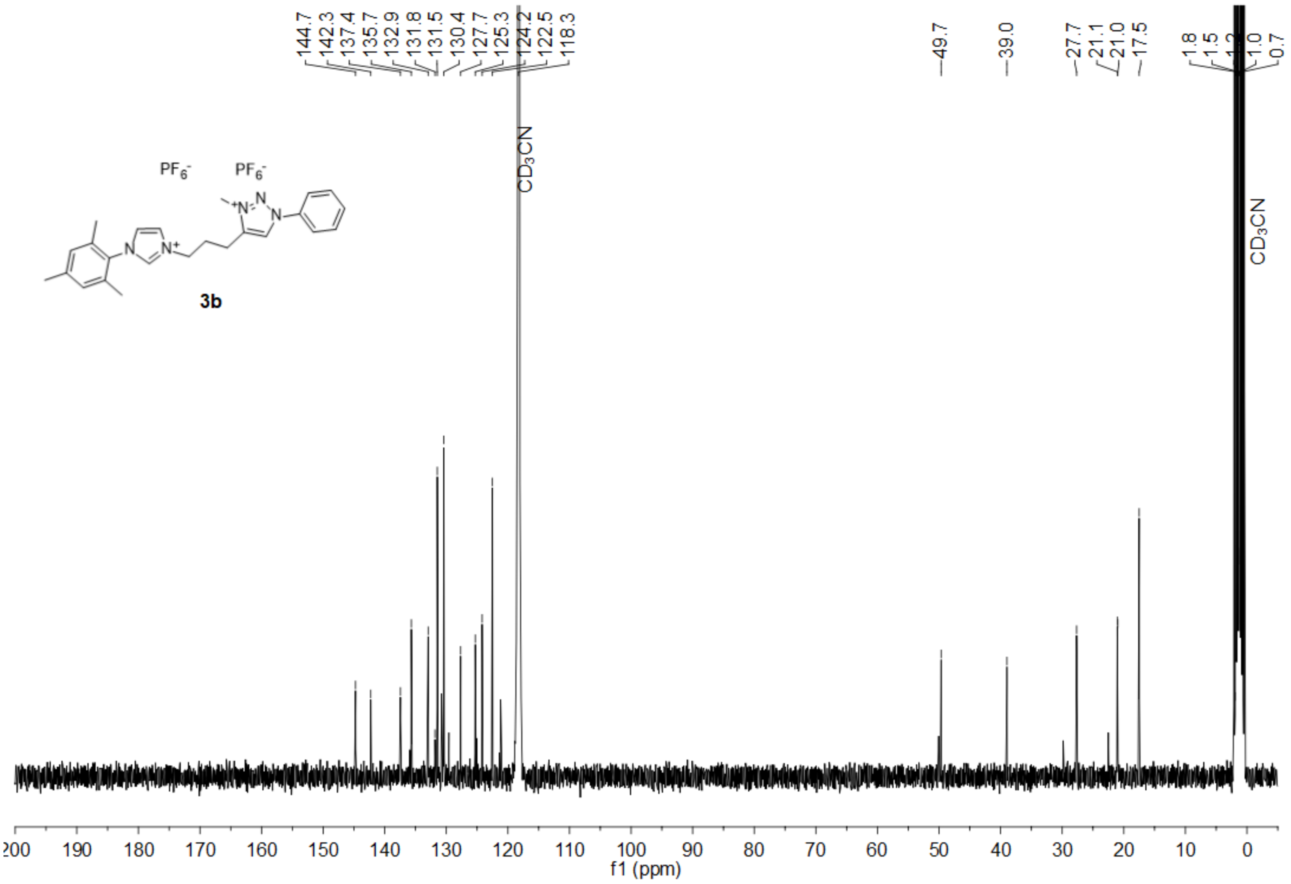

Figure S9. $^{13}C\{^{1}H\}$ NMR of compound **3b** in CD_3CN.

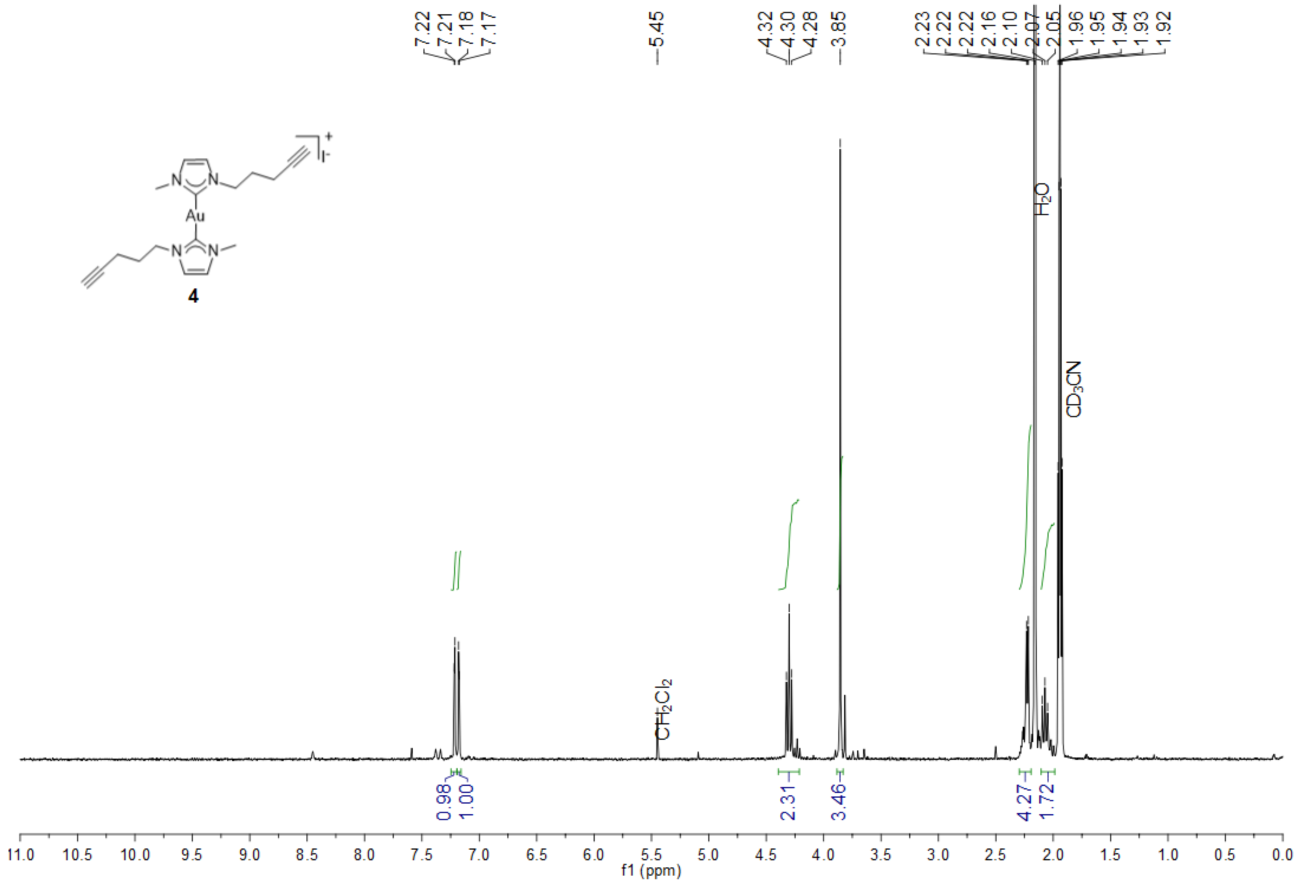

Figure S10. ^{1}H NMR of complex **4** in CD_3CN.

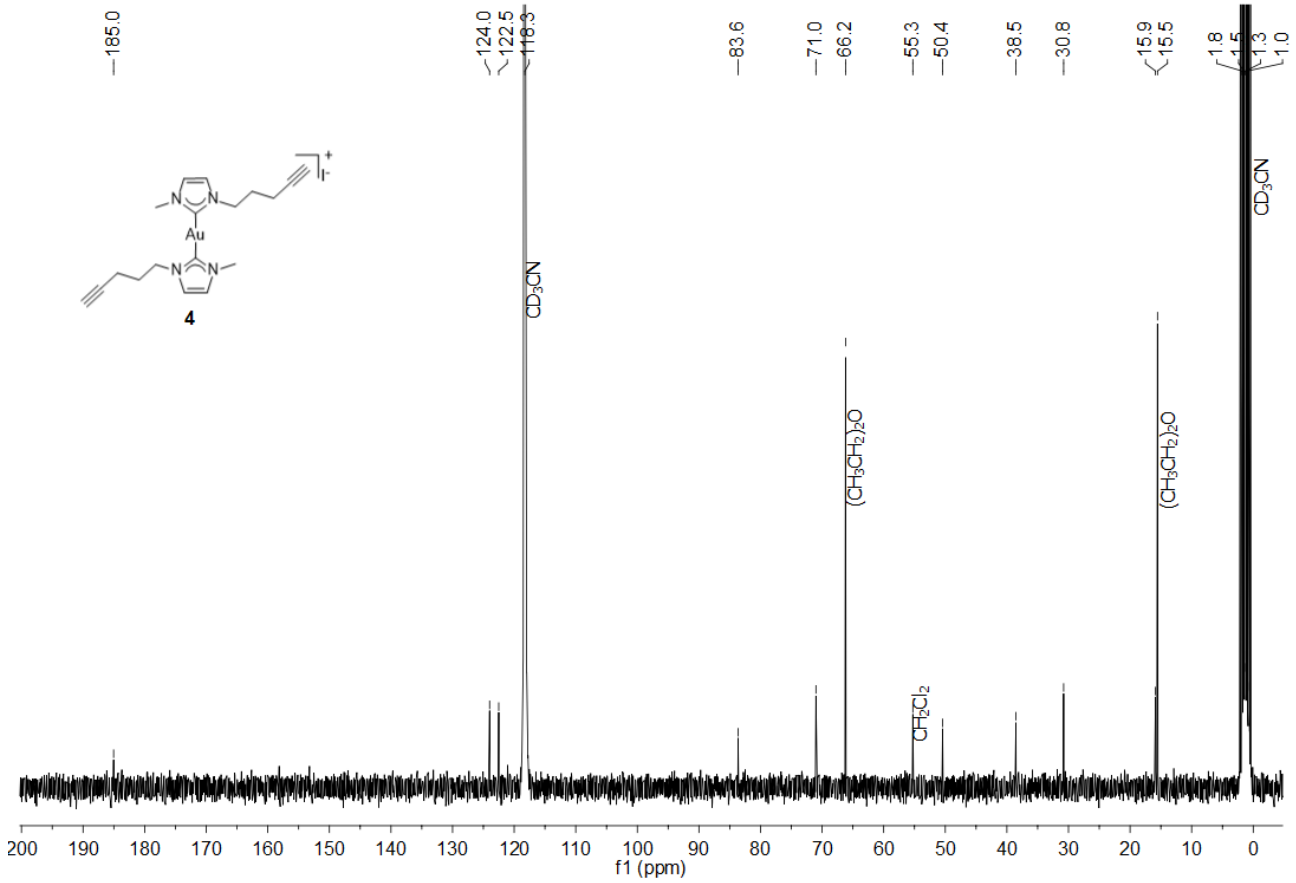

Figure S11. $^{13}C\{^1H\}$ NMR of complex **4** in CD_3CN.

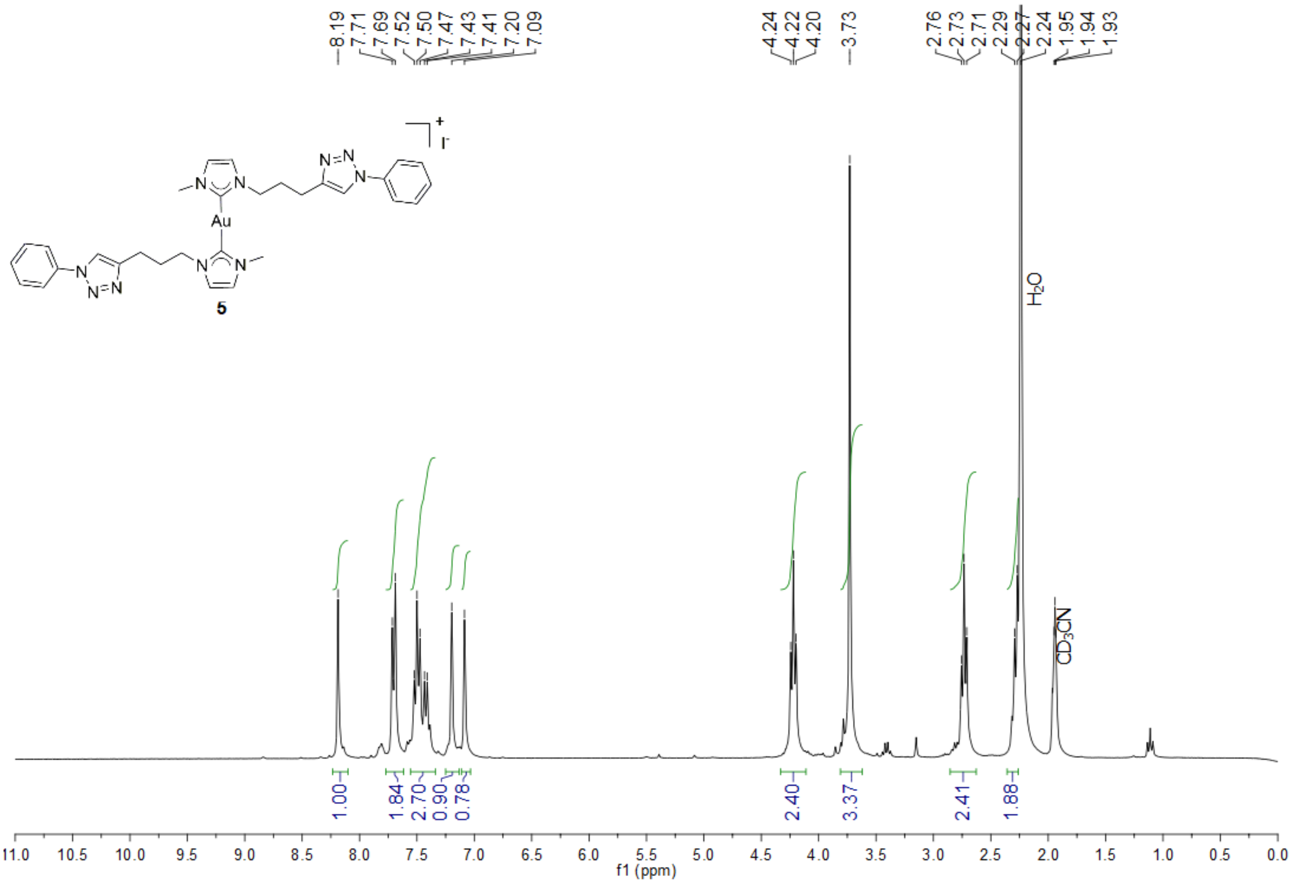

Figure S12. ^{1}H NMR of complex **5** in CD_3CN.

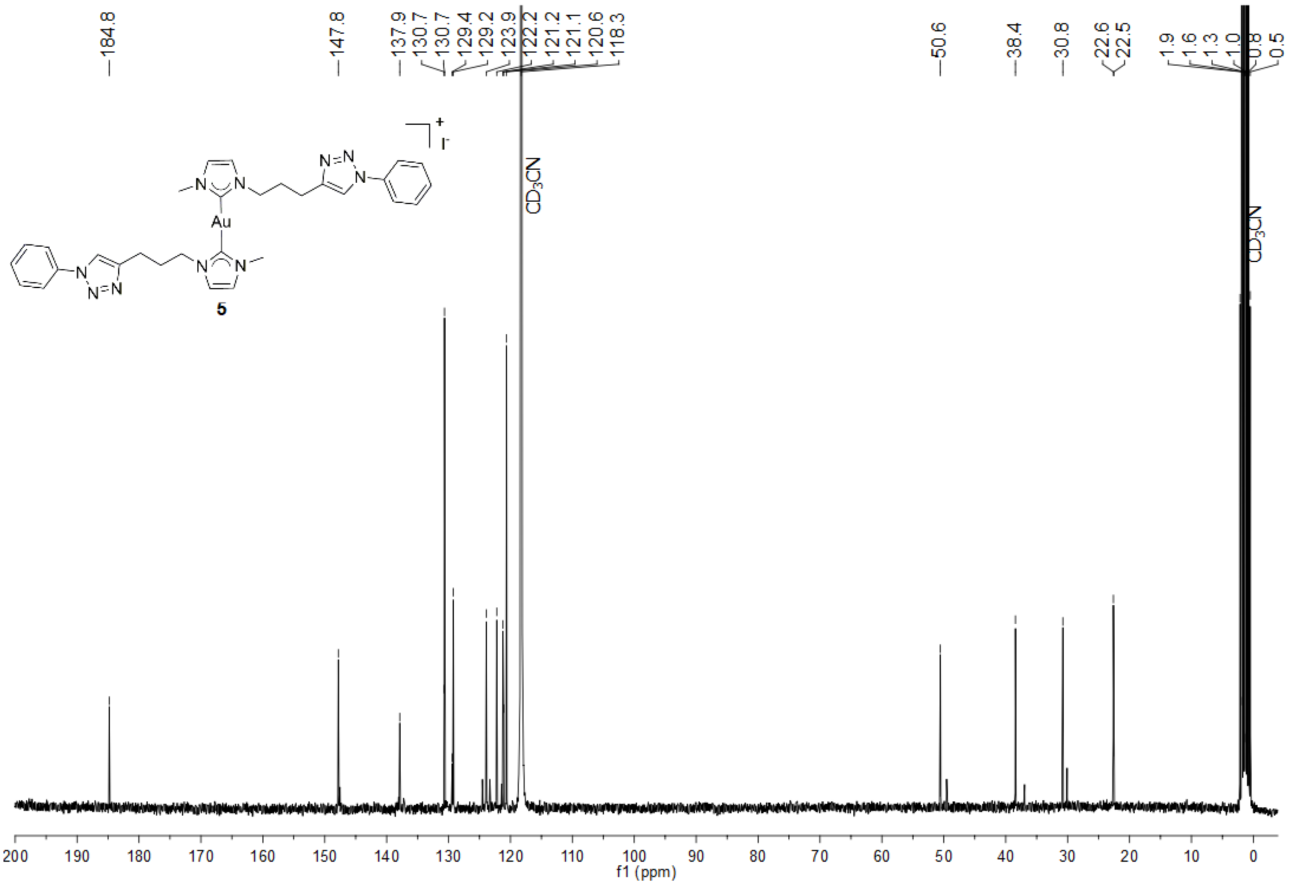

Figure S13. $^{13}C\{^{1}H\}$ NMR of complex **5** in CD_3CN.

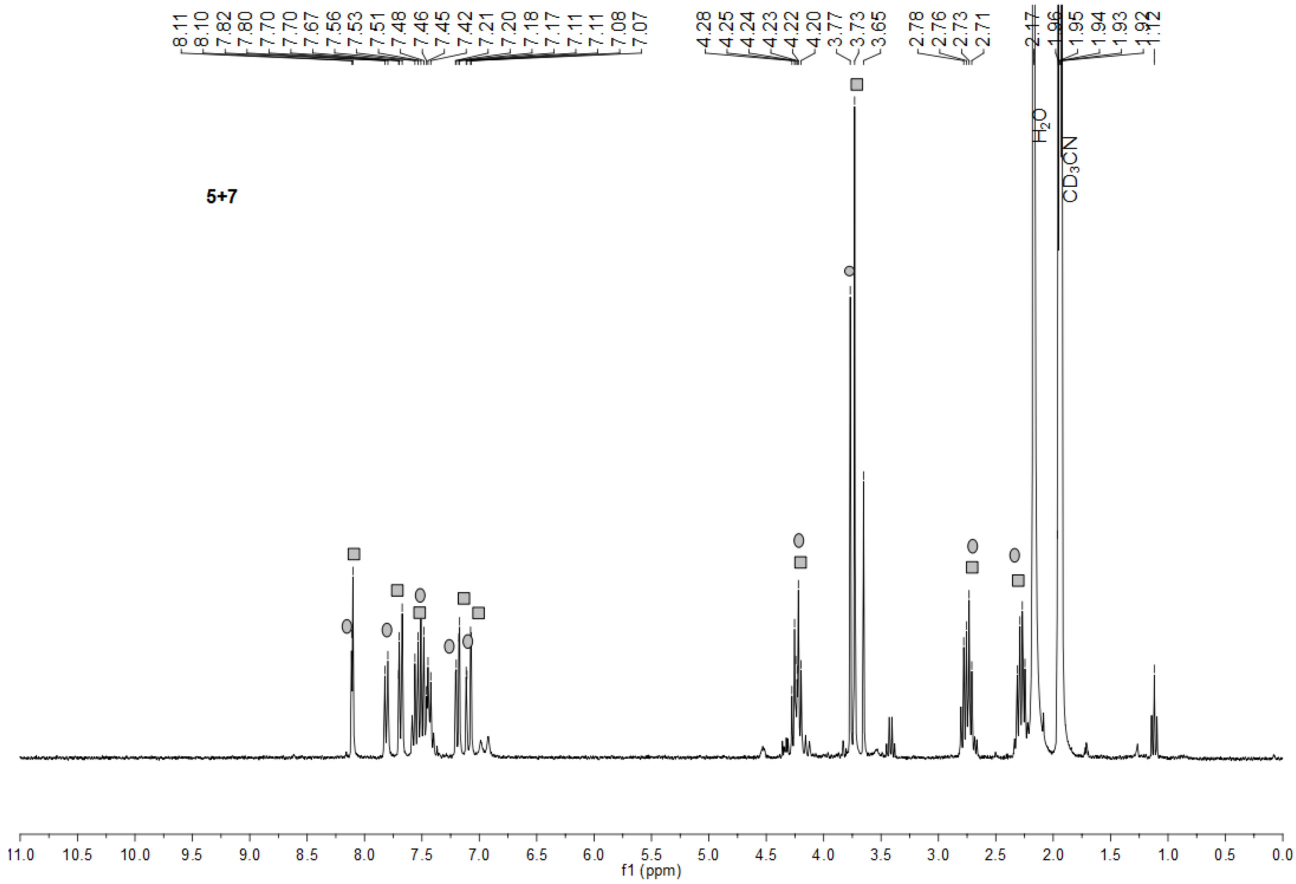

Figure S14. ^{1}H NMR of the mixture **5+7** in CD_3CN; ■ identifies signals of complex **5**, ● those of complex **7**.

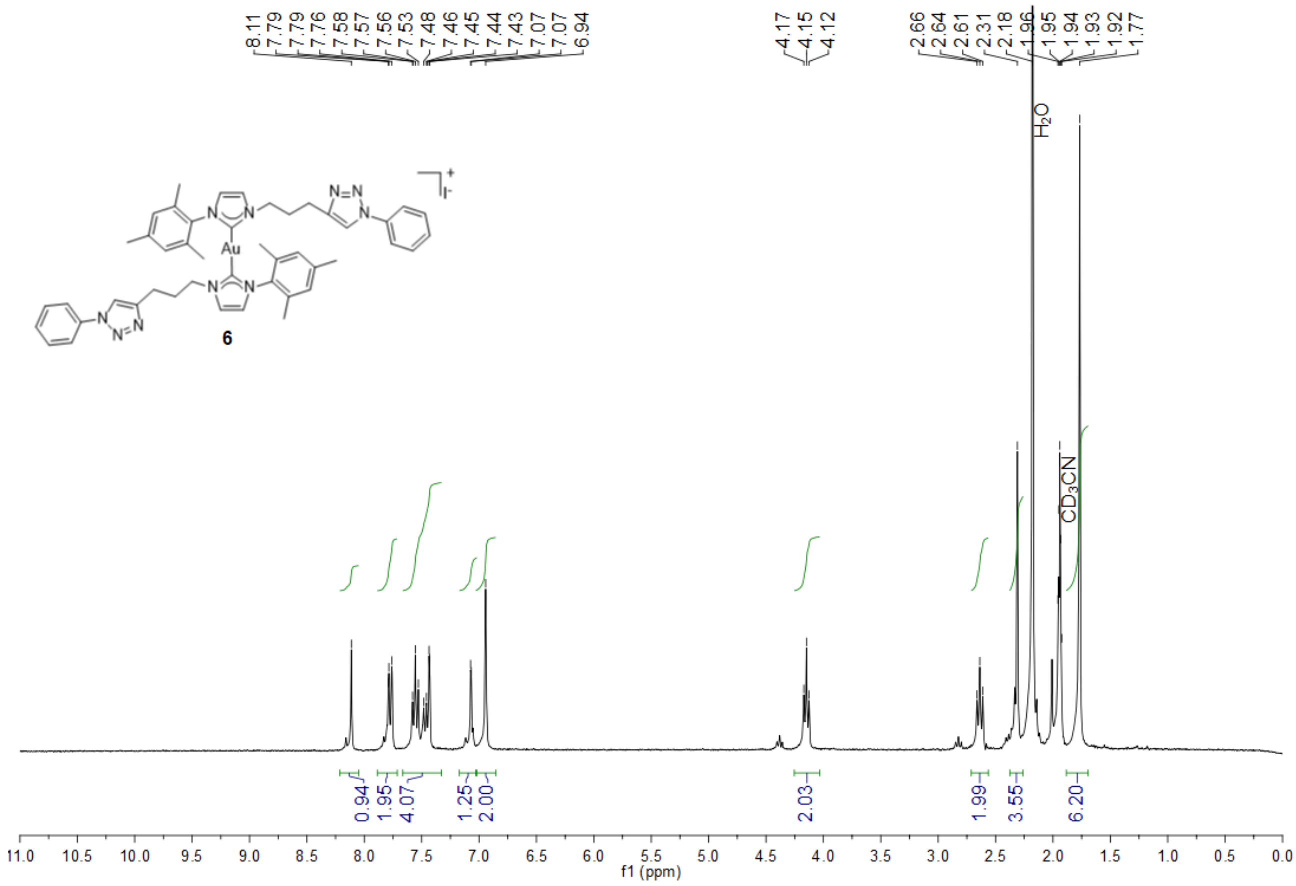

Figure S15. ^{1}H NMR of complex **6** in CD_3CN.

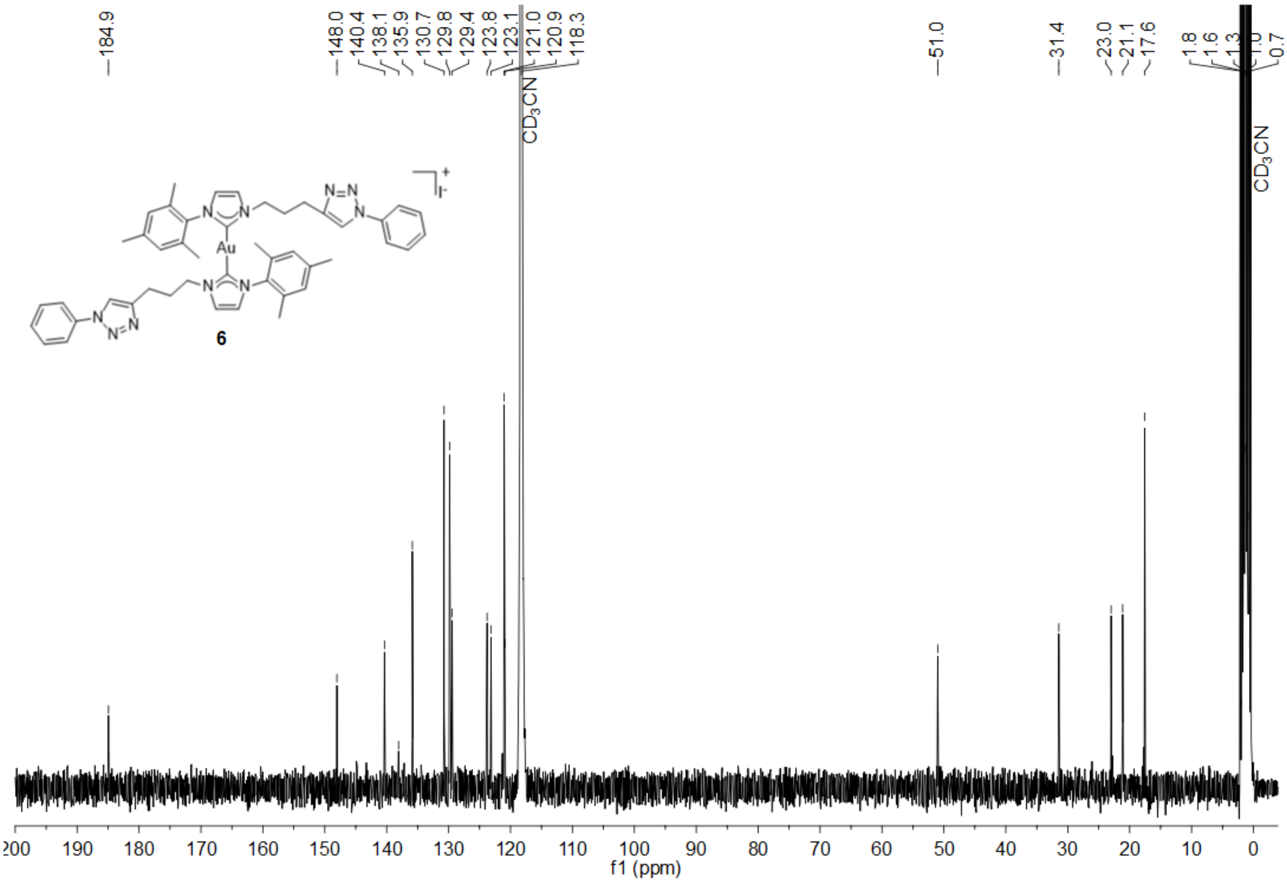

Figure S16. $^{13}C\{^{1}H\}$ NMR of complex **6** in CD_3CN.

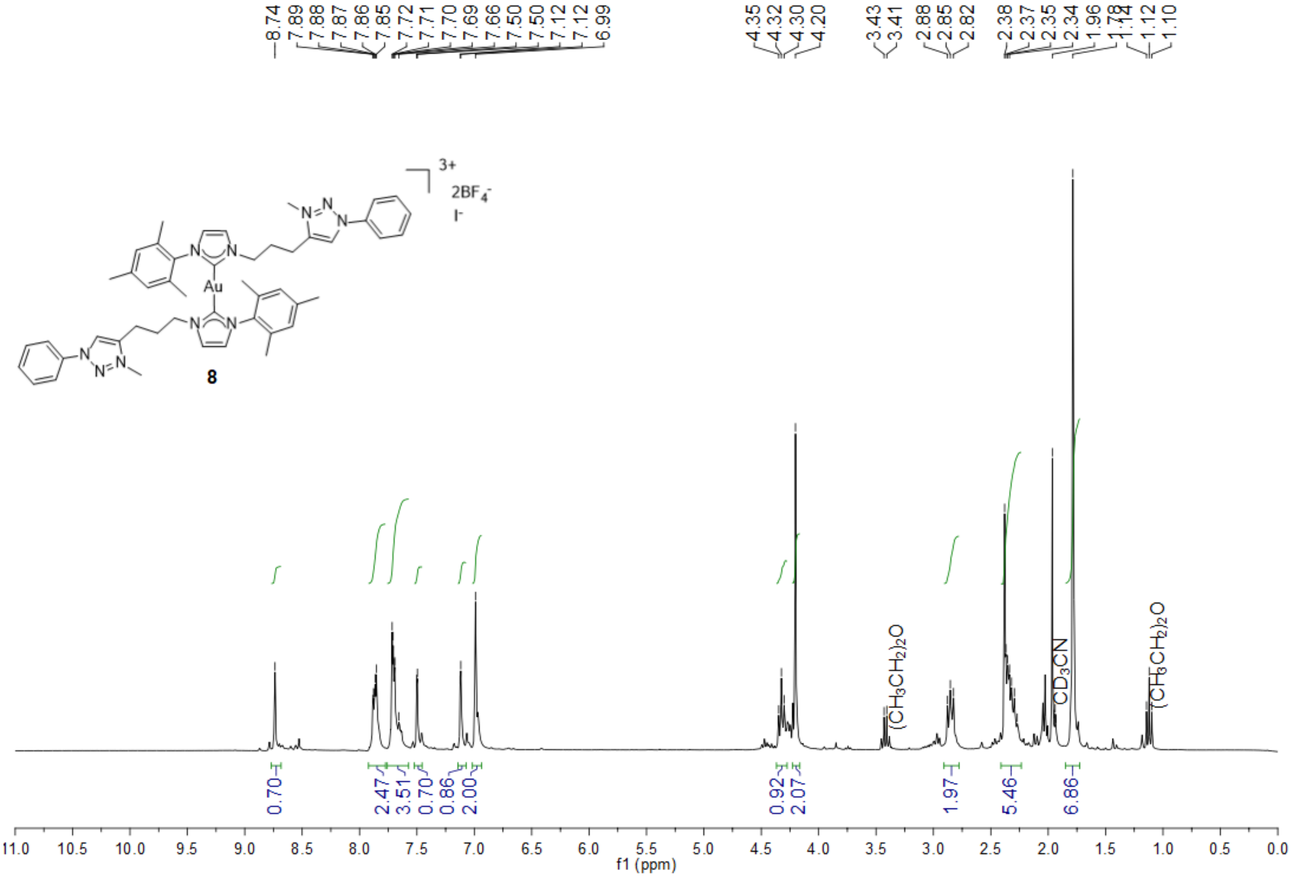

Figure S17. ^{1}H NMR of complex **8** in CD_3CN.

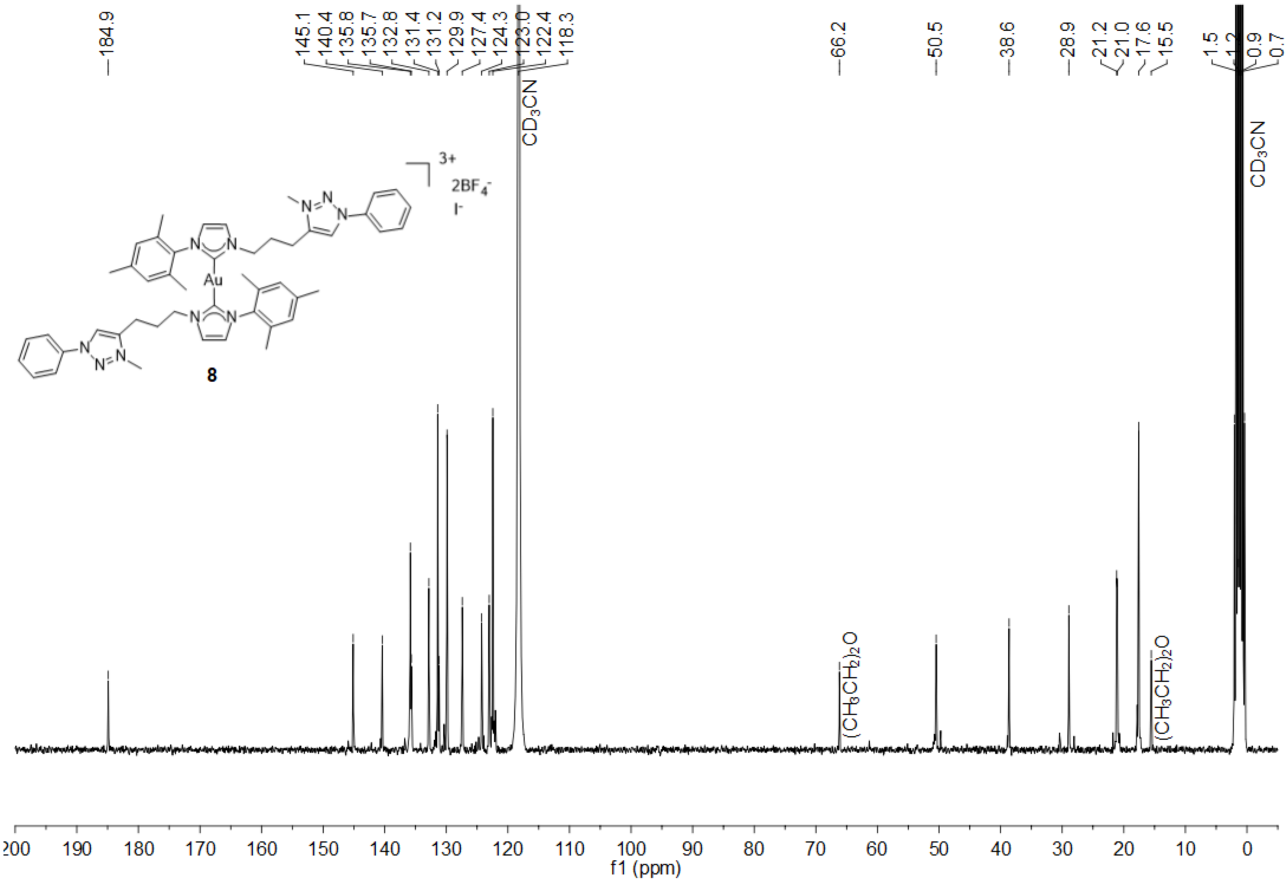

Figure S18. $^{13}C\{^{1}H\}$ NMR of complex **8** in CD_3CN.

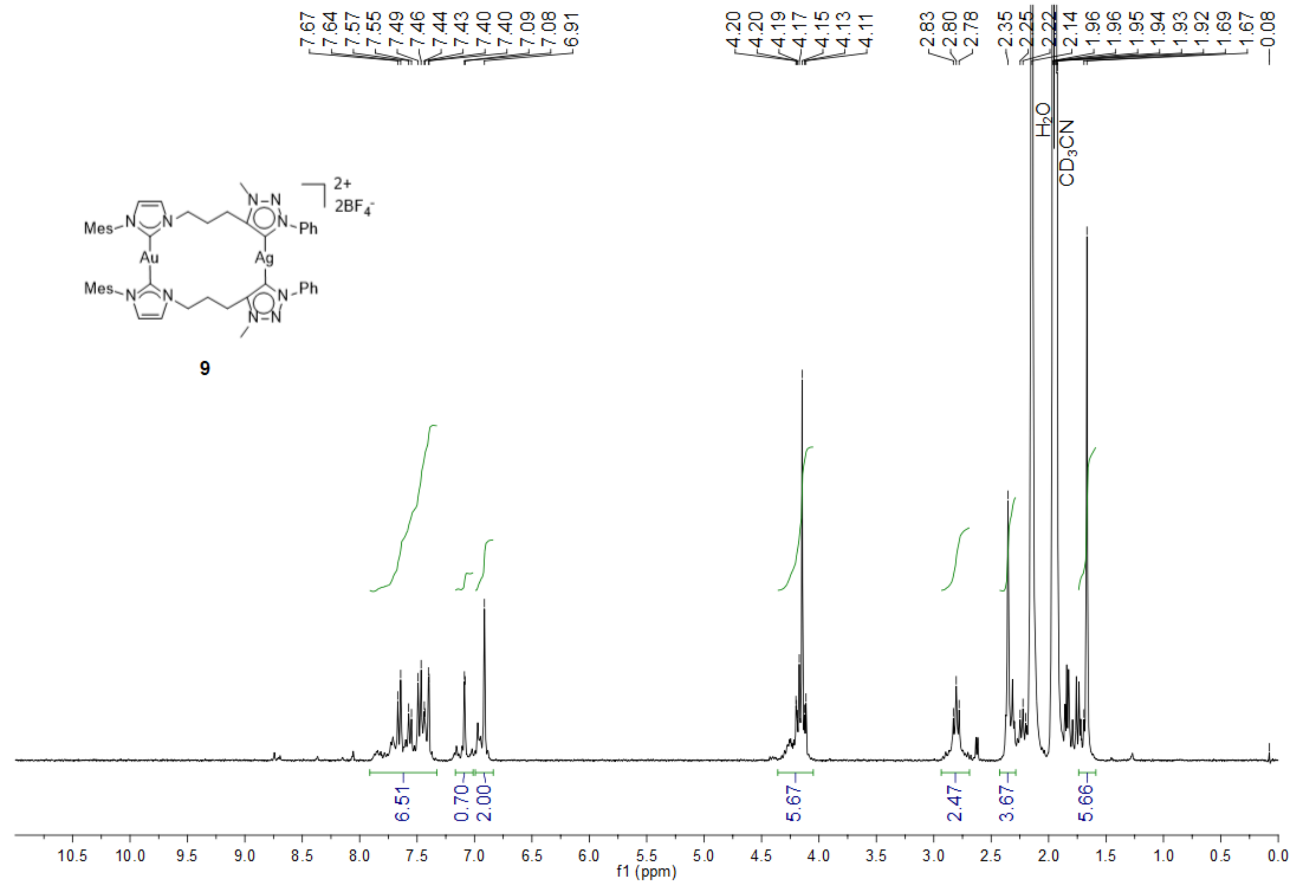

Figure S19. ^{1}H NMR of complex **9** in CD_3CN.

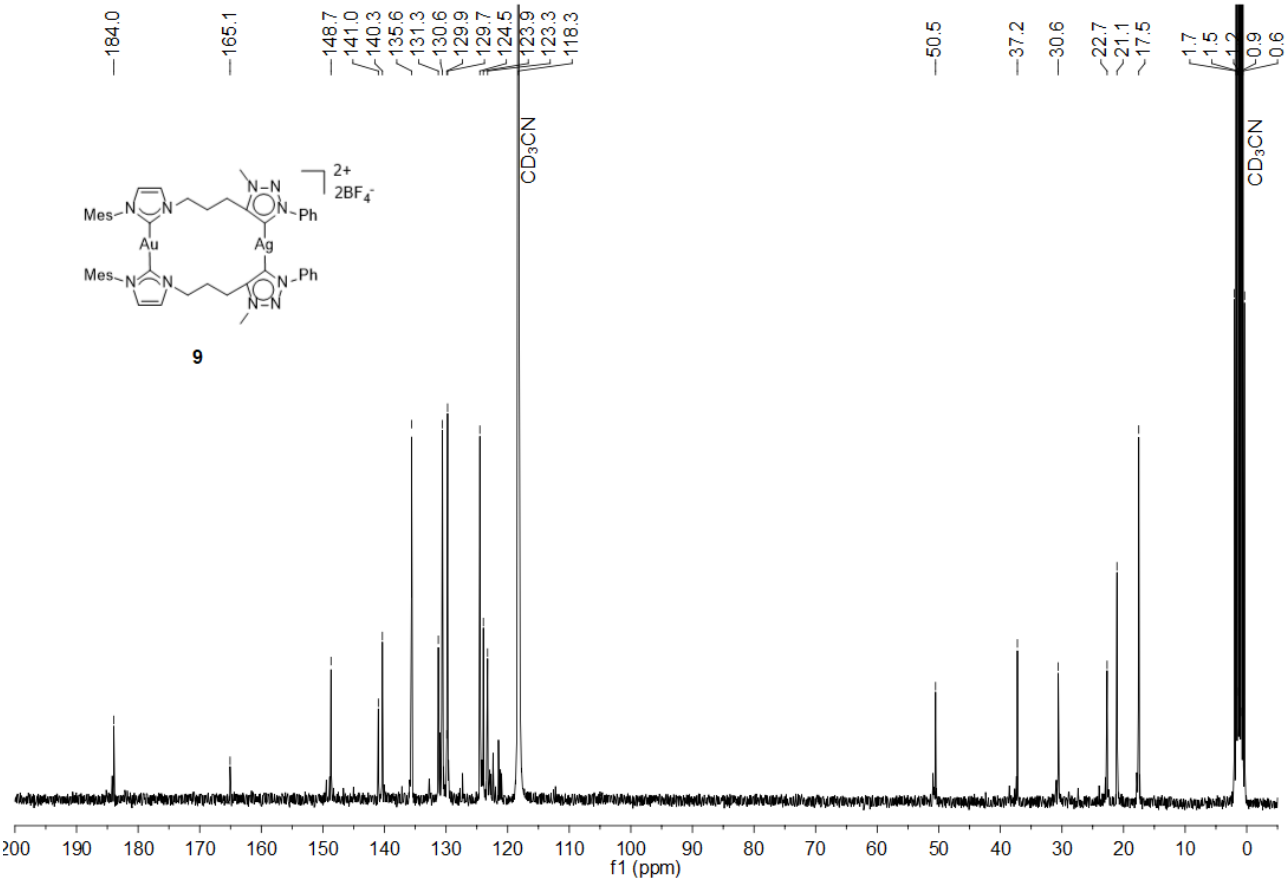

Figure S20. ${}^{13}C\{{}^{1}H\}$ NMR of complex **9** in CD_3CN.

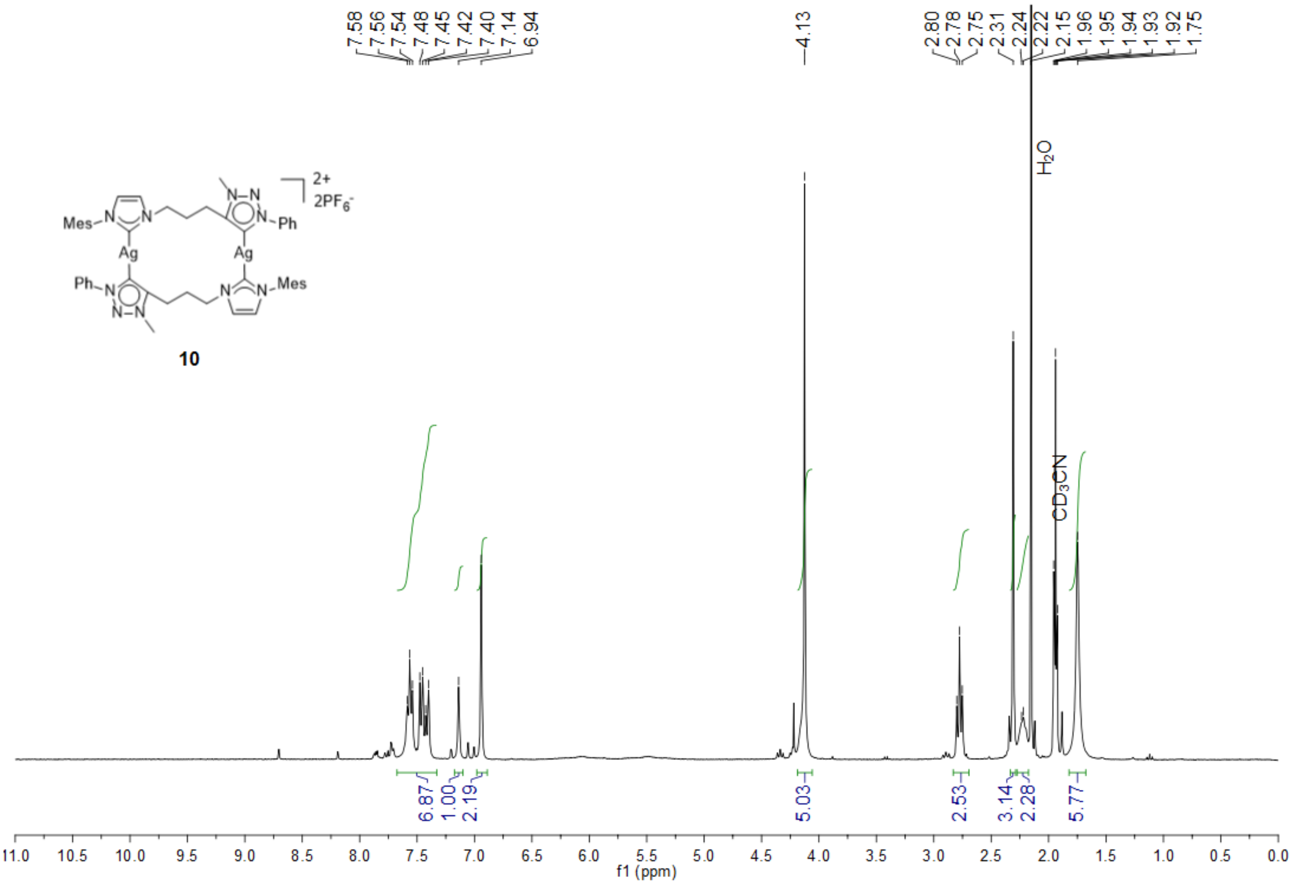

Figure S21. ${}^{1}H$ NMR of complex **10** in CD_3CN.

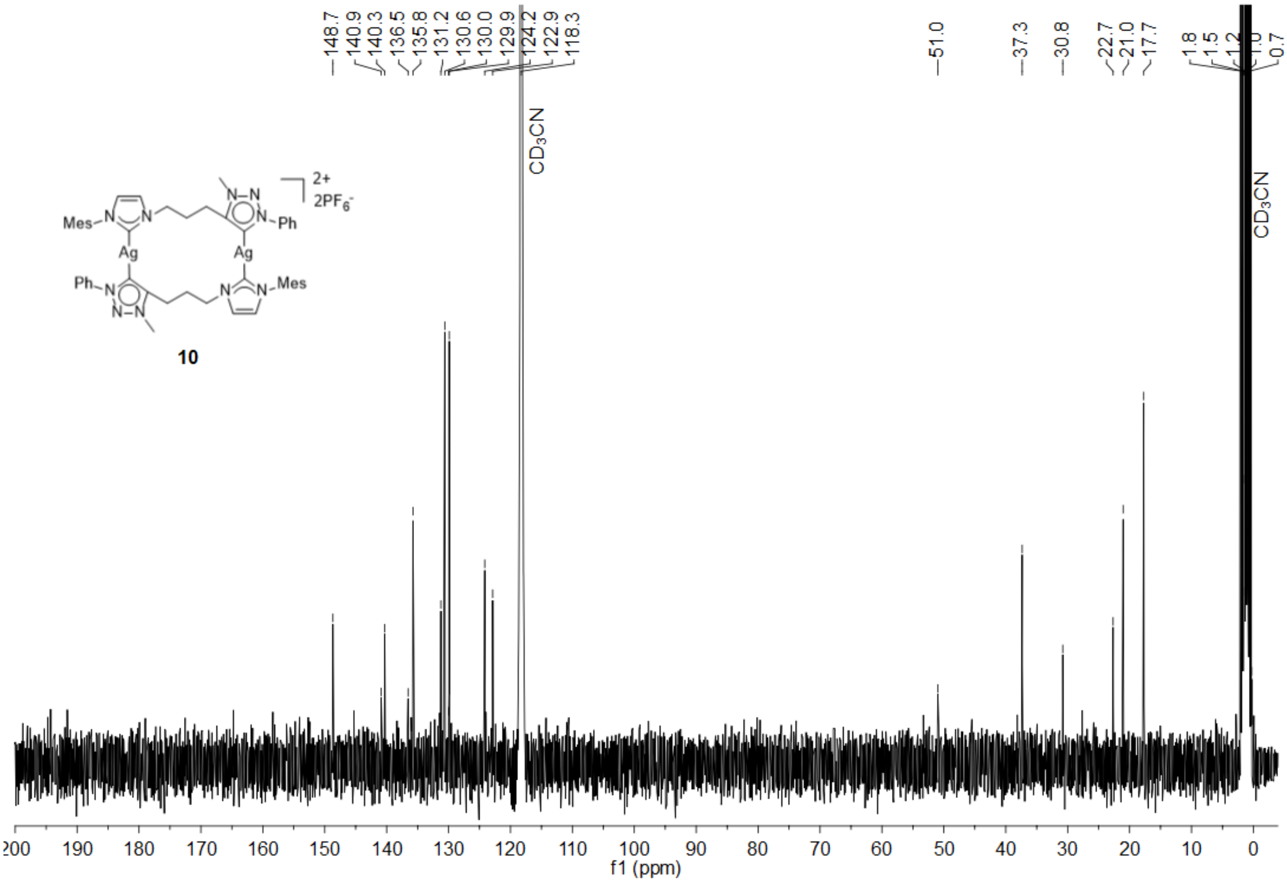

Figure S22 $^{13}C\{^1H\}$ NMR of complex**10** in CD_3CN.

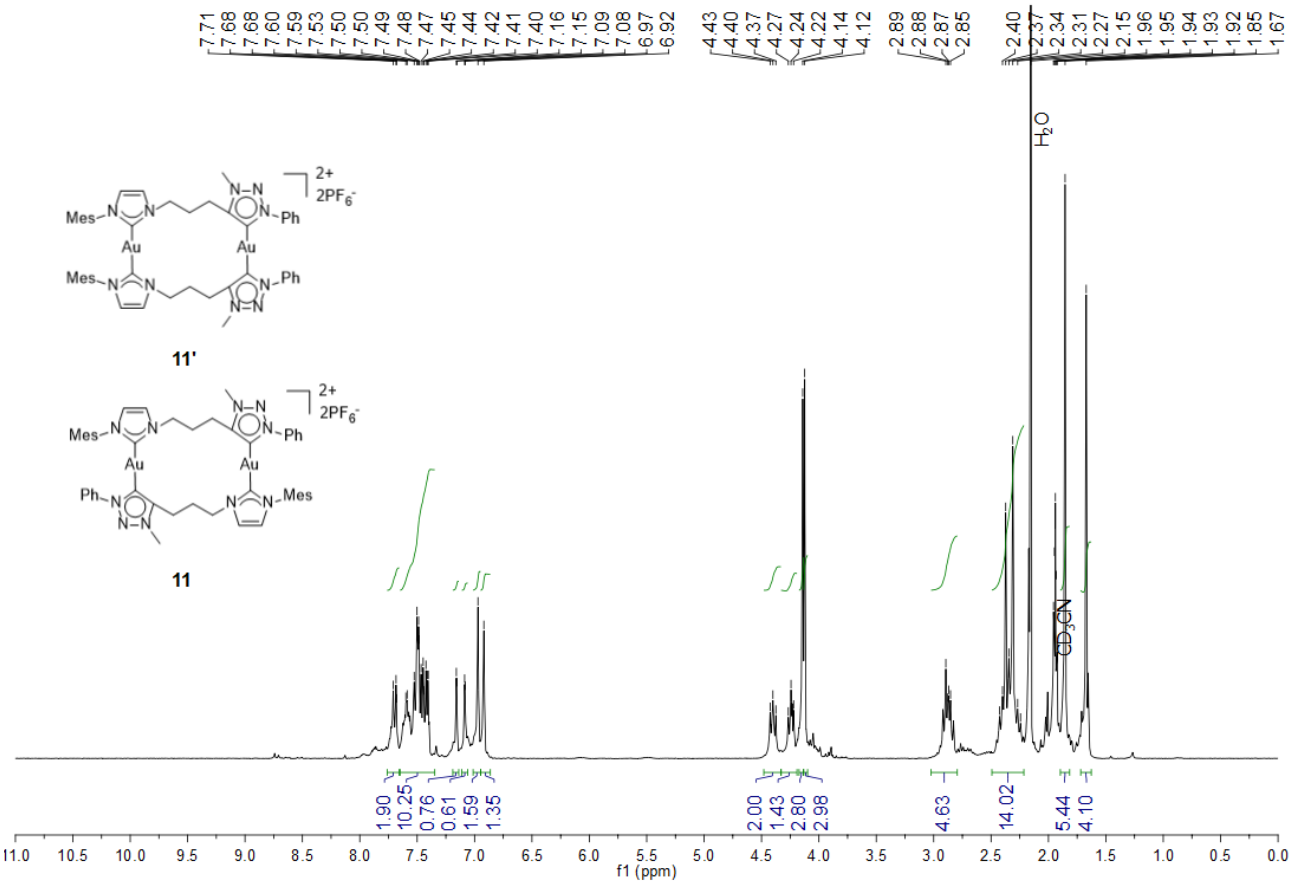

Figure S23. ^1H NMR of the mixture of complexes **11**/**11'** in CD_3CN.

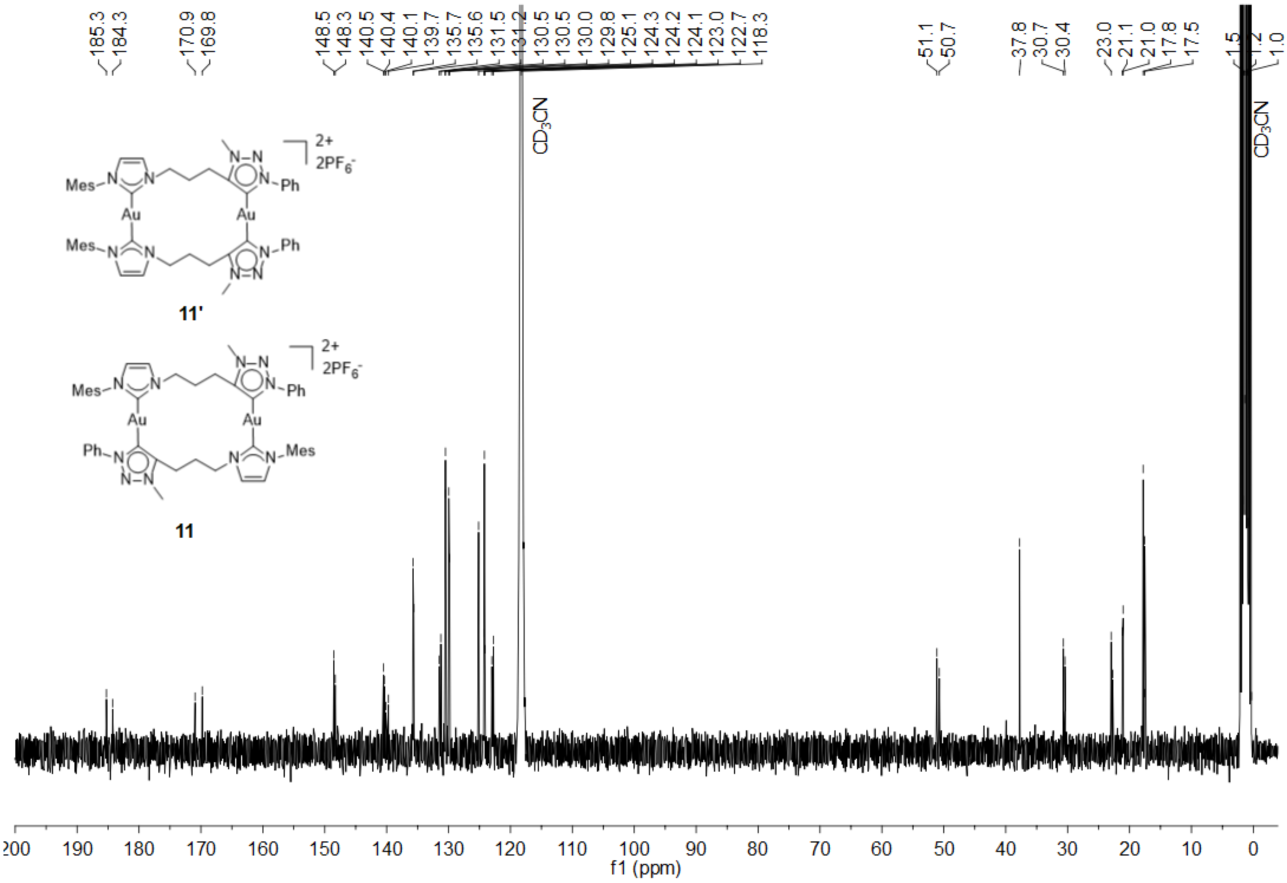

Figure S24. $^{13}C\{^1H\}$ NMR of the mixture of complexes **11/11'** in CD_3CN.

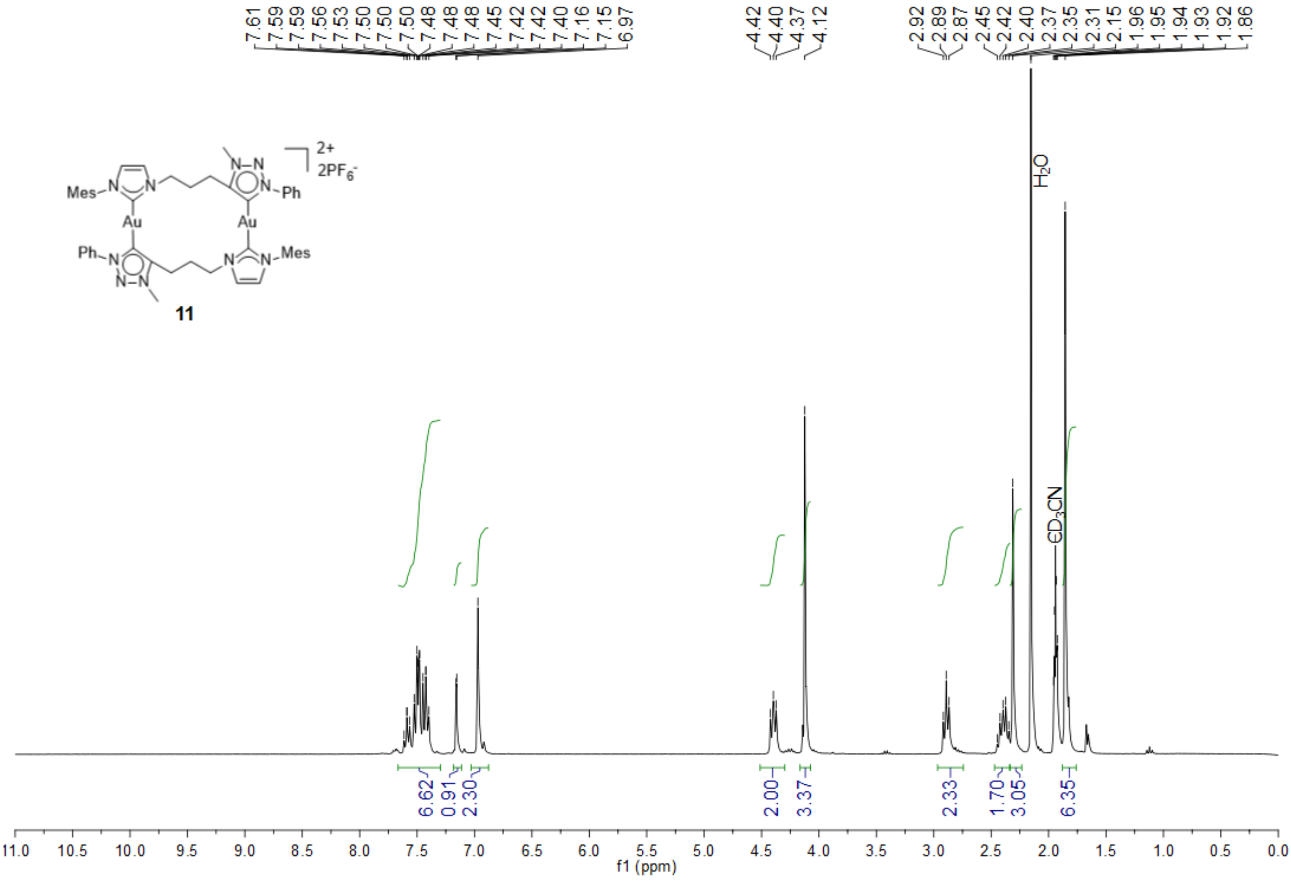

Figure S25. ^1H NMR of the complex **11** in CD_3CN.

Table S1. Crystal data and structure refinement for compounds **3b**, **4**, **6**, **7** and **11**

Compound	**3b**	**4**	**6**	**7**	**11**
Empirical formula	$C_{24}H_{29}F_{12}N_5P_2$	$C_{18}H_{24}AuIN_4$	$C_{46}H_{50}AuIN_{10}$	$C_{94}H_{108}Au_3I_9N_{32}$	$C_{48}H_{54}Au_2F_{12}N_{10}P_2$
Formula weight	677.46	620.28	1066.82	3419.12	1454.88
Temperature/K	294(7)	297.3(3)	298.9(6)	298(2)	297.3(4)
Crystal system	monoclinic	monoclinic	triclinic	triclinic	monoclinic
Space group	$P2_1/n$	$P2_1/n$	P-1	P-1	$P2_1/c$
a/Å	7.3780(3)	6.5131(3)	10.1224(4)	9.3633(2)	12.7531(6)
b/Å	28.1830(15)	13.8875(5)	14.3562(5)	14.5000(4)	8.4120(4)
c/Å	14.5586(7)	11.8617(5)	15.8637(6)	23.1244(7)	25.2975(10)
α/°	90	90	96.879(3)	106.078(2)	90
β/°	98.482(4)	103.920(4)	99.056(3)	91.868(2)	96.017(4)
γ/°	90	90	93.241(3)	108.675(2)	90
Volume/Å^3	2994.1(2)	1041.39(8)	2253.48(15)	2832.44(14)	2698.9(2)
Z	4	2	2	1	2
ρ_{calc}g/cm^3	1.503	1.978	1.572	2.004	1.790
μ/mm^{-1}	0.244	24.952	3.993	26.869	5.574
F(000)	1384.0	584.0	1056.0	1610.0	1416.0
Crystal size/mm^3	0.3 × 0.1 × 0.08	0.2 × 0.08 × 0.08	0.3 × 0.1 × 0.08	0.2 × 0.1 × 0.1	0.3 × 0.15 × 0.04
Radiation	MoKα (λ = 0.71073)	CuKα (λ = 1.54184)	MoKα (λ = 0.71073)	CuKα (λ = 1.54184)	Mo Kα (λ = 0.71073)
2Θ range for data collection/°	5.178 to 52.742	9.98 to 117.866	4.808 to 52.742	6.728 to 130.156	4.794 to 52.734
Reflections collected	12962	4470	17515	33736	29048
Independent reflections	6082	1492	9212	9336	5514
Data/restraints/parameters	6082/648/502	1492/0/113	9212/0/529	9336/520/629	5514/63/338
Goodness-of-fit on F^2	1.044	1.020	1.061	1.062	1.093
Final R indexes [I>=2σ (I)]	R_1 = 0.0572, wR_2 = 0.1491	R_1 = 0.0496, wR_2 = 0.1407	R_1 = 0.0337, wR_2 = 0.0877	R_1 = 0.0594, wR_2 = 0.1488	R_1 = 0.0330, wR_2 = 0.0785
Final R indexes [all data]	R_1 = 0.0744, wR_2 = 0.1628	R_1 = 0.0557, wR_2 = 0.1532	R_1 = 0.0379, wR_2 = 0.0908	R_1 = 0.0686, wR_2 = 0.1553	R_1 = 0.0392, wR_2 = 0.0818
Largest diff. peak/hole / e Å^{-3}	0.40/-0.28	1.24/-1.66	2.61/-1.34	3.08/-1.87	2.28/-0.95
CCDC	1903106	1903104	1903103	1903105	1903107
